# Supplementary material for: SARS-CoV-2 nonstructural protein 3 remodels the phosphorylation of target proteins via protein-protein interactions
Source: Microbiol Spectr. 2026 Mar 16;14(4):e02915-25. doi: 10.1128/spectrum.02915-25 (PMC13055223; doi:10.1128/spectrum.02915-25)

**Complete sequences of rSARS-CoV-2 mCherry-N:**

attaaaggtttataccttcccaggtaacaaaccaaccaactttcgatctcttgtagatctgttctctaaacgaactttaaaatctgtgtggctgtcactcggctgcatgcttagtgcactcacgcagtataattaataactaattactgtcgttgacaggacacgagtaactcgtctatcttctgcaggctgcttacggtttcgtccgtgttgcagccgatcatcagcacatctaggtttcgtccgggtgtgaccgaaaggtaagatggagagccttgtccctggtttcaacgagaaaacacacgtccaactcagtttgcctgttttacaggttcgcgacgtgctcgtacgtggctttggagactccgtggaggaggtcttatcagaggcacgtcaacatcttaaagatggcacttgtggcttagtagaagttgaaaaaggcgttttgcctcaacttgaacagccctatgtgttcatcaaacgttcggatgctcgaactgcacctcatggtcatgttatggttgagctggtagcagaactcgaaggcattcagtacggtcgtagtggtgagacacttggtgtccttgtccctcatgtgggcgaaataccagtggcttaccgcaaggttcttcttcgtaagaacggtaataaaggagctggtggccatagttacggcgccgatctaaagtcatttgacttaggcgacgagcttggcactgatccttatgaagattttcaagaaaactggaacactaaacatagcagtggtgttacccgtgaactcatgcgtgagcttaacggaggggcatacactcgctatgtcgataacaacttctgtggccctgatggctaccctcttgagtgcattaaagaccttctagcacgtgctggtaaagcttcatgcactttgtccgaacaactggactttattgacactaagaggggtgtatactgctgccgtgaacatgagcatgaaattgcttggtacacggaacgttctgaaaagagctatgaattgcagacaccttttgaaattaaattggcaaagaaatttgacaccttcaatggggaatgtccaaattttgtatttcccttaaattccataatcaagactattcaaccaagggttgaaaagaaaaagcttgatggctttatgggtagaattcgatctgtctatccagttgcgtcaccaaatgaatgcaaccaaatgtgcctttcaactctcatgaagtgtgatcattgtggtgaaacttcatggcagacgggcgattttgttaaagccacttgcgaattttgtggcactgagaatttgactaaagaaggtgccactacttgtggttacttaccccaaaatgctgttgttaaaatttattgtccagcatgtcacaattcagaagtaggacctgagcatagtcttgccgaataccataatgaatctggcttgaaaaccattcttcgtaagggtggtcgcactattgcctttggaggctgtgtgttctcttatgttggttgccataacaagtgtgcctattgggttccacgtgctagcgctaacataggttgtaaccatacaggtgttgttggagaaggttccgaaggtcttaatgacaaccttcttgaaatactccaaaaagagaaagtcaacatcaatattgttggtgactttaaacttaatgaagagatcgccattattttggcatctttttctgcttccacaagtgcttttgtggaaactgtgaaaggtttggattataaagcattcaaacaaattgttgaatcctgtggtaattttaaagttacaaaaggaaaagctaaaaaaggtgcctggaatattggtgaacagaaatcaatactgagtcctctttatgcatttgcatcagaggctgctcgtgttgtacgatcaattttctcccgcactcttgaaactgctcaaaattctgtgcgtgttttacagaaggccgctataacaatactagatggaatttcacagtattcactgagactcattgatgctatgatgttcacatctgatttggctactaacaatctagttgtaatggcctacattacaggtggtgttgttcagttgacttcgcagtggctaactaacatctttggcactgtttatgaaaaactcaaacccgtccttgattggcttgaagagaagtttaaggaaggtgtagagtttcttagagacggttgggaaattgttaaatttatctcaacctgtgcttgtgaaattgtcggtggacaaattgtcacctgtgcaaaggaaattaaggagagtgttcagacattctttaagcttgtaaataaatttttggctttgtgtgctgactctatcattattggtggagctaaacttaaagccttgaatttaggtgaaacatttgtcacgcactcaaagggattgtacagaaagtgtgttaaatccagagaagaaactggcctactcatgcctctaaaagccccaaaagaaattatcttcttagagggagaaacacttcccacagaagtgttaacagaggaagttgtcttgaaaactggtgatttacaaccattagaacaacctactagtgaagctgttgaagctccattggttggtacaccagtttgtattaacgggcttatgttgctcgaaatcaaagacacagaaaagtactgtgcccttgcacctaatatgatggtaacaaacaataccttcacactcaaaggcggtgcaccaacaaaggttacttttggtgatgacactgtgatagaagtgcaaggttacaagagtgtgaatatcacttttgaacttgatgaaaggattgataaagtacttaatgagaagtgctctgcctatacagttgaactcggtacagaagtaaatgagttcgcctgtgttgtggcagatgctgtcataaaaactttgcaaccagtatctgaattacttacaccactgggcattgatttagatgagtggagtatggctacatactacttatttgatgagtctggtgagtttaaattggcttcacatatgtattgttctttctaccctccagatgaggatgaagaagaaggtgattgtgaagaagaagagtttgagccatcaactcaatatgagtatggtactgaagatgattaccaaggtaaacctttggaatttggtgccacttctgctgctcttcaacctgaagaagagcaagaagaagattggttagatgatgatagtcaacaaactgttggtcaacaagacggcagtgaggacaatcagacaactactattcaaacaattgttgaggttcaacctcaattagagatggaacttacaccagttgttcagactattgaagtgaatagttttagtggttatttaaaacttactgacaatgtatacattaaaaatgcagacattgtggaagaagctaaaaaggtaaaaccaacagtggttgttaatgcagccaatgtttaccttaaacatggaggaggtgttgcaggagccttaaataaggctactaacaatgccatgcaagttgaatctgatgattacatagctactaatggaccacttaaagtgggtggtagttgtgttttaagcggacacaatcttgctaaacactgtcttcatgttgtcggcccaaatgttaacaaaggtgaagacattcaacttcttaagagtgcttatgaaaattttaatcagcacgaagttctacttgcaccattattatcagctggtatttttggtgctgaccctatacattctttaagagtttgtgtagatactgttcgcacaaatgtctacttagctgtctttgataaaaatctctatgacaaacttgtttcaagctttttggaaatgaagagtgaaaagcaagttgaacaaaagatcgctgagattcctaaagaggaagttaagccatttataactgaaagtaaaccttcagttgaacagagaaaacaagatgataagaaaatcaaagcttgtgttgaagaagttacaacaactctggaagaaactaagttcctcacagaaaacttgttactttatattgacattaatggcaatcttcatccagattctgccactcttgttagtgacattgacatcactttcttaaagaaagatgctccatatatagtgggtgatgttgttcaagagggtgttttaactgctgtggttatacctactaaaaaggctggtggcactactgaaatgctagcgaaagctttgagaaaagtgccaacagacaattatataaccacttacccgggtcagggtttaaatggttacactgtagaggaggcaaagacagtgcttaaaaagtgtaaaagtgccttttacattctaccatctattatctctaatgagaagcaagaaattcttggaactgtttcttggaatttgcgagaaatgcttgcacatgcagaagaaacacgcaaattaatgcctgtctgtgtggaaactaaagccatagtttcaactatacagcgtaaatataagggtattaaaatacaagagggtgtggttgattatggtgctagattttacttttacaccagtaaaacaactgtagcgtcacttatcaacacacttaacgatctaaatgaaactcttgttacaatgccacttggctatgtaacacatggcttaaatttggaagaagctgctcggtatatgagatctctcaaagtgccagctacagtttctgtttcttcacctgatgctgttacagcgtataatggttatcttacttcttcttctaaaacacctgaagaacattttattgaaaccatctcacttgctggttcctataaagattggtcctattctggacaatctacacaactaggtatagaatttcttaagagaggtgataaaagtgtatattacactagtaatcctaccacattccacctagatggtgaagttatcacctttgacaatcttaagacacttctttctttgagagaagtgaggactattaaggtgtttacaacagtagacaacattaacctccacacgcaagttgtggacatgtcaatgacatatggacaacagtttggtccaacttatttggatggagctgatgttactaaaataaaacctcataattcacatgaaggtaaaacattttatgttttacctaatgatgacactctacgtgttgaggcttttgagtactaccacacaactgatcctagttttctgggtaggtacatgtcagcattaaatcacactaaaaagtggaaatacccacaagttaatggtttaacttctattaaatgggcagataacaactgttatcttgccactgcattgttaacactccaacaaatagagttgaagtttaatccacctgctctacaagatgcttattacagagcaagggctggtgaagctgctaacttttgtgcacttatcttagcctactgtaataagacagtaggtgagttaggtgatgttagagaaacaatgagttacttgtttcaacatgccaatttagattcttgcaaaagagtcttgaacgtggtgtgtaaaacttgtggacaacagcagacaacccttaagggtgtagaagctgttatgtacatgggcacactttcttatgaacaatttaagaaaggtgttcagataccttgtacgtgtggtaaacaagctacaaaatatctagtacaacaggagtcaccttttgttatgatgtcagcaccacctgctcagtatgaacttaagcatggtacatttacttgtgctagtgagtacactggtaattaccagtgtggtcactataaacatataacttctaaagaaactttgtattgcatagacggtgctttacttacaaagtcctcagaatacaaaggtcctattacggatgttttctacaaagaaaacagttacacaacaaccataaaaccagttacttataaattggatggtgttgtttgtacagaaattgaccctaagttggacaattattataagaaagacaattcttatttcacagagcaaccaattgatcttgtaccaaaccaaccatatccaaacgcaagcttcgataattttaagtttgtatgtgataatatcaaatttgctgatgatttaaaccagttaactggttataagaaacctgcttcaagagagcttaaagttacatttttccctgacttaaatggtgatgtggtggctattgattataaacactacacaccctcttttaagaaaggagctaaattgttacataaacctattgtttggcatgttaacaatgcaactaataaagccacgtataaaccaaatacctggtgtatacgttgtctttggagcacaaaaccagttgaaacatcaaattcgtttgatgtactgaagtcagaggacgcgcagggaatggataatcttgcctgcgaagatctaaaaccagtctctgaagaagtagtggaaaatcctaccatacagaaagacgttcttgagtgtaatgtgaaaactaccgaagttgtaggagacattatacttaaaccagcaaataatagtttaaaaattacagaagaggttggccacacagatctaatggctgcttatgtagacaattctagtcttactattaagaaacctaatgaattatctagagtattaggtttgaaaacccttgctactcatggtttagctgctgttaatagtgtcccttgggatactatagctaattatgctaagccttttcttaacaaagttgttagtacaactactaacatagttacacggtgtttaaaccgtgtttgtactaattatatgccttatttctttactttattgctacaattgtgtacttttactagaagtacaaattctagaattaaagcatctatgccgactactatagcaaagaatactgttaagagtgtcggtaaattttgtctagaggcttcatttaattatttgaagtcacctaatttttctaaactgataaatattataatttggtttttactattaagtgtttgcctaggttctttaatctactcaaccgctgctttaggtgttttaatgtctaatttaggcatgccttcttactgtactggttacagagaaggctatttgaactctactaatgtcactattgcaacctactgtactggttctataccttgtagtgtttgtcttagtggtttagattctttagacacctatccttctttagaaactatacaaattaccatttcatcttttaaatgggatttaactgcttttggcttagttgcagagtggtttttggcatatattcttttcactaggtttttctatgtacttggattggctgcaatcatgcaattgtttttcagctattttgcagtacattttattagtaattcttggcttatgtggttaataattaatcttgtacaaatggccccgatttcagctatggttagaatgtacatcttctttgcatcattttattatgtatggaaaagttatgtgcatgttgtagacggttgtaattcatcaacttgtatgatgtgttacaaacgtaatagagcaacaagagtcgaatgtacaactattgttaatggtgttagaaggtccttttatgtctatgctaatggaggtaaaggcttttgcaaactacacaattggaattgtgttaattgtgatacattctgtgctggtagtacatttattagtgatgaagttgcgagagacttgtcactacagtttaaaagaccaataaatcctactgaccagtcttcttacatcgttgatagtgttacagtgaagaatggttccatccatctttactttgataaagctggtcaaaagacttatgaaagacattctctctctcattttgttaacttagacaacctgagagctaataacactaaaggttcattgcctattaatgttatagtttttgatggtaaatcaaaatgtgaagaatcatctgcaaaatcagcgtctgtttactacagtcagcttatgtgtcaacctatactgttactagatcaggcattagtgtctgatgttggtgatagtgcggaagttgcagttaaaatgtttgatgcttacgttaatacgttttcatcaacttttaacgtaccaatggaaaaactcaaaacactagttgcaactgcagaagctgaacttgcaaagaatgtgtccttagacaatgtcttatctacttttatttcagcagctcggcaagggtttgttgattcagatgtagaaactaaagatgttgttgaatgtcttaaattgtcacatcaatctgacatagaagttactggcgatagttgtaataactatatgctcacctataacaaagttgaaaacatgacaccccgtgaccttggtgcttgtattgactgtagtgcgcgtcatattaatgcgcaggtagcaaaaagtcacaacattgctttgatatggaacgttaaagatttcatgtcattgtctgaacaactacgaaaacaaatacgtagtgctgctaaaaagaataacttaccttttaagttgacatgtgcaactactagacaagttgttaatgttgtaacaacaaagatagcacttaagggtggtaaaattgttaataattggttgaagcagttaattaaagttacacttgtgttcctttttgttgctgctattttctatttaataacacctgttcatgtcatgtctaaacatactgacttttcaagtgaaatcataggatacaaggctattgatggtggtgtcactcgtgacatagcatctacagatacttgttttgctaacaaacatgctgattttgacacatggtttagtcagcgtggtggtagttatactaatgacaaagcttgcccattgattgctgcagtcataacaagagaagtgggttttgtcgtgcctggtttgcctggcacgatattacgcacaactaatggtgactttttgcatttcttacctagagtttttagtgcagttggtaacatctgttacacaccatcaaaacttatagagtacactgactttgcaacatcagcttgtgttttggctgctgaatgtacaatttttaaagatgcttctggtaagccagtaccatattgttatgataccaatgtactagaaggttctgttgcttatgaaagtttacgccctgacacacgttatgtgctcatggatggctctattattcaatttcctaacacctaccttgaaggttctgttagagtggtaacaacttttgattctgagtactgtaggcacggcacttgtgaaagatcagaagctggtgtttgtgtatctactagtggtagatgggtacttaacaatgattattacagatctttaccaggagttttctgtggtgtagatgctgtaaatttacttactaatatgtttacaccactaattcaacctattggtgctttggacatatcagcatctatagtagctggtggtattgtagctatcgtagtaacatgccttgcctactattttatgaggtttagaagagcttttggtgaatacagtcatgtagttgcctttaatactttactattccttatgtcattcactgtactctgtttaacaccagtttactcattcttacctggtgtttattctgttatttacttgtacttgacattttatcttactaatgatgtttcttttttagcacatattcagtggatggttatgttcacacctttagtacctttctggataacaattgcttatatcatttgtatttccacaaagcatttctattggttctttagtaattacctaaagagacgtgtagtctttaatggtgtttcctttagtacttttgaagaagctgcgctgtgcacctttttgttaaataaagaaatgtatctaaagttgcgtagtgatgtgctattacctcttacgcaatataatagatacttagctctttataataagtacaagtattttagtggagcaatggatacaactagctacagagaagctgcttgttgtcatctcgcaaaggctctcaatgacttcagtaactcaggttctgatgttctttaccaaccaccacaaacctctatcacctcagctgttttgcagagtggttttagaaaaatggcattcccatctggtaaagttgagggttgtatggtacaagtaacttgtggtacaactacacttaacggtctttggcttgatgacgtagtttactgtccaagacatgtgatctgcacctctgaagacatgcttaaccctaattatgaagatttactcattcgtaagtctaatcataatttcttggtacaggctggtaatgttcaactcagggttattggacattctatgcaaaattgtgtacttaagcttaaggttgatacagccaatcctaagacacctaagtataagtttgttcgcattcaaccaggacagactttttcagtgttagcttgttacaatggttcaccatctggtgtttaccaatgtgctatgaggcccaatttcactattaagggttcattccttaatggttcatgtggtagtgttggttttaacatagattatgactgtgtctctttttgttacatgcaccatatggaattaccaactggagttcatgctggcacagacttagaaggtaacttttatggaccttttgttgacaggcaaacagcacaagcagctggtacggacacaactattacagttaatgttttagcttggttgtacgctgctgttataaatggagacaggtggtttctcaatcgatttaccacaactcttaatgactttaaccttgtggctatgaagtacaattatgaacctctaacacaagaccatgttgacatactaggacctctttctgctcaaactggaattgccgttttagatatgtgtgcttcattaaaagaattactgcaaaatggtatgaatggacgtaccatattgggtagtgctttattagaagatgaatttacaccttttgatgttgttagacaatgctcaggtgttactttccaaagtgcagtgaaaagaacaatcaagggtacacaccactggttgttactcacaattttgacttcacttttagttttagtccagagtactcaatggtctttgttcttttttttgtatgaaaatgcctttttaccttttgctatgggtattattgctatgtctgcttttgcaatgatgtttgtcaaacataagcatgcatttctctgtttgtttttgttaccttctcttgccactgtagcttattttaatatggtctatatgcctgctagttgggtgatgcgtattatgacatggttggatatggttgatactagtttgtctggttttaagctaaaagactgtgttatgtatgcatcagctgtagtgttactaatccttatgacagcaagaactgtgtatgatgatggtgctaggagagtgtggacacttatgaatgtcttgacactcgtttataaagtttattatggtaatgctttagatcaagccatttccatgtgggctcttataatctctgttacttctaactactcaggtgtagttacaactgtcatgtttttggccagaggtattgtttttatgtgtgttgagtattgccctattttcttcataactggtaatacacttcagtgtataatgctagtttattgtttcttaggctatttttgtacttgttactttggcctcttttgtttactcaaccgctactttagactgactcttggtgtttatgattacttagtttctacacaggagtttagatatatgaattcacagggactactcccacccaagaatagcatagatgccttcaaactcaacattaaattgttgggtgttggtggcaaaccttgtatcaaagtagccactgtacagtctaaaatgtcagatgtaaagtgcacatcagtagtcttactctcagttttgcaacaactcagagtagaatcatcatctaaattgtgggctcaatgtgtccagttacacaatgacattctcttagctaaagatactactgaagcctttgaaaaaatggtttcactactttctgttttgctttccatgcagggtgctgtagacataaacaagctttgtgaagaaatgctggacaacagggcaaccttacaagctatagcctcagagtttagttcccttccatcatatgcagcttttgctactgctcaagaagcttatgagcaggctgttgctaatggtgattctgaagttgttcttaaaaagttgaagaagtctttgaatgtggctaaatctgaatttgaccgtgatgcagccatgcaacgtaagttggaaaagatggctgatcaagctatgacccaaatgtataaacaggctagatctgaggacaagagggcaaaagttactagtgctatgcagacaatgcttttcactatgcttagaaagttggataatgatgcactcaacaacattatcaacaatgcaagagatggttgtgttcccttgaacataatacctcttacaacagcagccaaactaatggttgtcataccagactataacacatataaaaatacgtgtgatggtacaacatttacttatgcatcagcattgtgggaaatccaacaggttgtagatgcagatagtaaaattgttcaacttagtgaaattagtatggacaattcacctaatttagcatggcctcttattgtaacagctttaagggccaattctgctgtcaaattacagaataatgagcttagtcctgttgcactacgacagatgtcttgtgctgccggtactacacaaactgcttgcactgatgacaatgcgttagcttactacaacacaacaaagggaggtaggtttgtacttgcactgttatccgatttacaggatttgaaatgggctagattccctaagagtgatggaactggtactatctatacagaactggaaccaccttgtaggtttgttacagacacacctaaaggtcctaaagtgaagtatttatactttattaaaggattaaacaacctaaatagaggtatggtacttggtagtttagctgccacagtacgtctacaagctggtaatgcaacagaagtgcctgccaattcaactgtattatctttctgtgcttttgctgtagatgctgctaaagcttacaaagattatctagctagtgggggacaaccaatcactaattgtgttaagatgttgtgtacacacactggtactggtcaggcaataacagttacaccggaagccaatatggatcaagaatcctttggtggtgcatcgtgttgtctgtactgccgttgccacatagatcatccaaatcctaaaggattttgtgacttaaaaggtaagtatgtacaaatacctacaacttgtgctaatgaccctgtgggttttacacttaaaaacacagtctgtaccgtctgcggtatgtggaaaggttatggctgtagttgtgatcaactccgcgaacccatgcttcagtcagctgatgcacaatcgtttttaaacgggtttgcggtgtaagtgcagcccgtcttacaccgtgcggcacaggcactagtactgatgtcgtatacagggcttttgacatctacaatgataaagtagctggttttgctaaattcctaaaaactaattgttgtcgcttccaagaaaaggacgaagatgacaatttaattgattcttactttgtagttaagagacacactttctctaactaccaacatgaagaaacaatttataatttacttaaggattgtccagctgttgctaaacatgacttctttaagtttagaatagacggtgacatggtaccacatatatcacgtcaacgtcttactaaatacacaatggcagacctcgtctatgctttaaggcattttgatgaaggtaattgtgacacattaaaagaaatacttgtcacatacaattgttgtgatgatgattatttcaataaaaaggactggtatgattttgtagaaaacccagatatattacgcgtatacgccaacttaggtgaacgtgtacgccaagctttgttaaaaacagtacaattctgtgatgccatgcgaaatgctggtattgttggtgtactgacattagataatcaagatctcaatggtaactggtatgatttcggtgatttcatacaaaccacgccaggtagtggagttcctgttgtagattcttattattcattgttaatgcctatattaaccttgaccagggctttaactgcagagtcacatgttgacactgacttaacaaagccttacattaagtgggatttgttaaaatatgacttcacggaagagaggttaaaactctttgaccgttattttaaatattgggatcagacataccacccaaattgtgttaactgtttggatgacagatgcattctgcattgtgcaaactttaatgttttattctctacagtgttcccacctacaagttttggaccactagtgagaaaaatatttgttgatggtgttccatttgtagtttcaactggataccacttcagagagctaggtgttgtacataatcaggatgtaaacttacatagctctagacttagttttaaggaattacttgtgtatgctgctgaccctgctatgcacgctgcttctggtaatctattactagataaacgcactacgtgcttttcagtagctgcacttactaacaatgttgcttttcaaactgtcaaacccggtaattttaacaaagacttctatgactttgctgtgtctaagggtttctttaaggaaggaagttctgttgaattaaaacacttcttctttgctcaggatggtaatgctgctatcagcgattatgactactatcgttataatctaccaacaatgtgtgatatcagacaactactatttgtagttgaagttgttgataagtactttgattgttacgatggtggctgtattaatgctaaccaagtcatcgtcaacaacctagacaaatcagctggttttccatttaataaatggggtaaggctagactttattatgattcaatgagttatgaggatcaagatgcacttttcgcatatacaaaacgtaatgtcatccctactataactcaaatgaatcttaagtatgccattagtgcaaagaatagagctcgcaccgtagctggtgtctctatctgtagtactatgaccaatagacagtttcatcaaaaattattgaaatcaatagccgccactagaggagctactgtagtaattggaacaagcaaattctatggtggttggcacaacatgttaaaaactgtttatagtgatgtagaaaaccctcaccttatgggttgggattatcctaaatgtgatagagccatgcctaacatgcttagaattatggcctcacttgttcttgctcgcaaacatacaacgtgttgtagcttgtcacaccgtttctatagattagctaatgagtgtgctcaagtattgagtgaaatggtcatgtgtggcggttcactatatgttaaaccaggtggaacctcatcaggagatgccacaactgcttatgctaatagtgtttttaacatttgtcaagctgtcacggccaatgttaatgcacttttatctactgatggtaacaaaattgccgataagtatgtccgcaatttacaacacagactttatgagtgtctctatagaaatagagatgttgacacagactttgtgaatgagttttacgcatatttgcgtaaacatttctcaatgatgatactctctgacgatgctgttgtgtgtttcaatagcacttatgcatctcaaggtctagtggctagcataaagaactttaagtcagttctttattatcaaaacaatgtttttatgtctgaagcaaaatgttggactgagactgaccttactaaaggacctcatgaattttgctctcaacatacaatgctagttaaacagggtgatgattatgtgtaccttccttacccagatccatcaagaatcctaggggccggctgttttgtagatgatatcgtaaaaacagatggtacacttatgattgaacggttcgtgtctttagctatagatgcttacccacttactaaacatcctaatcaggagtatgctgatgtctttcatttgtacttacaatacataagaaagctacatgatgagttaacaggacacatgttagacatgtattctgttatgcttactaatgataacacttcaaggtattgggaacctgagttttatgaggctatgtacacaccgcatacagtcttacaggctgttggggcttgtgttctttgcaattcacagacttcattaagatgtggtgcttgcatacgtagaccattcttatgttgtaaatgctgttacgaccatgtcatatcaacatcacataaattagtcttgtctgttaatccgtatgtttgcaatgctccaggttgtgatgtcacagatgtgactcaactttacttaggaggtatgagctattattgtaaatcacataaaccacccattagttttccattgtgtgctaatggacaagtttttggtttatataaaaatacatgtgttggtagcgataatgttactgactttaatgcaattgcaacatgtgactggacaaatgctggtgattacattttagctaacacctgtactgaaagactcaagctttttgcagcagaaacgctcaaagctactgaggagacatttaaactgtcttatggtattgctactgtacgtgaagtgctgtctgacagagaattacatctttcatgggaagttggtaaacctagaccaccacttaaccgaaattatgtctttactggttatcgtgtaactaaaaacagtaaagtacaaataggagagtacacctttgaaaaaggtgactatggtgatgctgttgtttaccgaggtacaacaacttacaaattaaatgttggtgattattttgtgctgacatcacatacagtaatgccattaagtgcacctacactagtgccacaagagcactatgttagaattactggcttatacccaacactcaatatctcagatgagttttctagcaatgttgcaaattatcaaaaggttggtatgcaaaagtattctacactccagggaccacctggtactggtaagagtcattttgctattggcctagctctctactacccttctgctcgcatagtgtatacagcttgctctcatgccgctgttgatgcactatgtgagaaggcattaaaatatttgcctatagataaatgtagtagaattatacctgcacgtgctcgtgtagagtgttttgataaattcaaagtgaattcaacattagaacagtatgtcttttgtactgtaaatgcattgcctgagacgacagcagatatagttgtctttgatgaaatttcaatggccacaaattatgatttgagtgttgtcaatgccagattacgtgctaagcactatgtgtacattggcgaccctgctcaattacctgcaccacgcacattgctaactaagggcacactagaaccagaatatttcaattcagtgtgtagacttatgaaaactataggtccagacatgttcctcggaacttgtcggcgttgtcctgctgaaattgttgacactgtgagtgctttggtttatgataataagcttaaagcacataaagacaaatcagctcaatgctttaaaatgttttataagggtgttatcacgcatgatgtttcatctgcaattaacaggccacaaataggcgtggtaagagaattccttacacgtaaccctgcttggagaaaagctgtctttatttcaccttataattcacagaatgctgtagcctcaaagattttgggactaccaactcaaactgttgattcatcacagggctcagaatatgactatgtcatattcactcaaaccactgaaacagctcactcttgtaatgtaaacagatttaatgttgctattaccagagcaaaagtaggcatactttgcataatgtctgatagagacctttatgacaagttgcaatttacaagtcttgaaattccacgtaggaatgtggcaactttacaagctgaaaatgtaacaggactttttaaagattgtagtaaggtaatcactgggttacatcctacacaggcacctacacacctcagtgttgacactaaattcaaaactgaaggtttatgtgttgacatacctggcatacctaaggacatgacctatagaagactcatctctatgatgggttttaaaatgaattatcaagttaatggttaccctaacatgtttatcacccgcgaagaagctataagacatgtacgtgcatggattggcttcgatgtcgaggggtgtcatgctactagagaagctgttggtaccaatttacctttacagctaggtttttctacaggtgttaacctagttgctgtacctacaggttatgttgatacacctaataatacagatttttccagagttagtgctaaaccaccgcctggagatcaatttaaacacctcataccacttatgtacaaaggacttccttggaatgtagtgcgtataaagattgtacaaatgttaagtgacacacttaaaaatctctctgacagagtcgtatttgtcttatgggcacatggctttgagttgacatctatgaagtattttgtgaaaataggacctgagcgcacctgttgtctatgtgatagacgtgccacatgcttttccactgcttcagacacttatgcctgttggcatcattctattggatttgattacgtctataatccgtttatgattgatgttcaacaatggggttttacaggtaacctacaaagcaaccatgatctgtattgtcaagtccatggtaatgcacatgtagctagttgtgatgcaatcatgactaggtgtctagctgtccacgagtgctttgttaagcgtgttgactggactattgaatatcctataattggtgatgaactgaagattaatgcggcttgtagaaaggttcaacacatggttgttaaagctgcattattagcagacaaattcccagttcttcacgacattggtaaccctaaagctattaagtgtgtacctcaagctgatgtagaatggaagttctatgatgcacagccttgtagtgacaaagcttataaaatagaagaattattctattcttatgccacacattctgacaaattcacagatggtgtatgcctattttggaattgcaatgtcgatagatatcctgctaattccattgtttgtagatttgacactagagtgctatctaaccttaacttgcctggttgtgatggtggcagtttgtatgtaaataaacatgcattccacacaccagcttttgataaaagtgcttttgttaatttaaaacaattaccatttttctattactctgacagtccatgtgagtctcatggaaaacaagtagtgtcagatatagattatgtaccactaaagtctgctacgtgtataacacgttgcaatttaggtggtgctgtctgtagacatcatgctaatgagtacagattgtatctcgatgcttataacatgatgatctcagctggctttagcttgtgggtttacaaacaatttgatacttataacctctggaacacttttacaagacttcagagtttagaaaatgtggcttttaatgttgtaaataagggacactttgatggacaacagggtgaagtaccagtttctatcattaataacactgtttacacaaaagttgatggtgttgatgtagaattgtttgaaaataaaacaacattacctgttaatgtagcatttgagctttgggctaagcgcaacattaaaccagtaccagaggtgaaaatactcaataatttgggtgtggacattgctgctaatactgtgatctgggactacaaaagagatgctccagcacatatatctactattggtgtttgttctatgactgacatagccaagaaaccaactgaaacgatttgtgcaccactcactgtcttttttgatggtagagttgatggtcaagtagacttatttagaaatgcccgtaatggtgttcttattacagaaggtagtgttaaaggtttacaaccatctgtaggtcccaaacaagctagtcttaatggagtcacattaattggagaagccgtaaaaacacagttcaattattataagaaagttgatggtgttgtccaacaattacctgaaacttactttactcagagtagaaatttacaagaatttaaacccaggagtcaaatggaaattgatttcttagaattagctatggatgaattcattgaacggtataaattagaaggctatgccttcgaacatatcgtttatggagattttagtcatagtcagttaggtggtttacatctactgattggactagctaaacgttttaaggaatcaccttttgaattagaagattttattcctatggacagtacagttaaaaactatttcataacagatgcgcaaacaggttcatctaagtgtgtgtgttctgttattgatttattacttgatgattttgttgaaataataaaatcccaagatttatctgtagtttctaaggttgtcaaagtgactattgactatacagaaatttcatttatgctttggtgtaaagatggccatgtagaaacattttacccaaaattacaatctagtcaagcgtggcaaccgggtgttgctatgcctaatctttacaaaatgcaaagaatgctattagaaaagtgtgaccttcaaaattatggtgatagtgcaacattacctaaaggcataatgatgaatgtcgcaaaatatactcaactgtgtcaatatttaaacacattaacattagctgtaccctataatatgagagttatacattttggtgctggttctgataaaggagttgcaccaggtacagctgttttaagacagtggttgcctacgggtacgctgcttgtcgattcagatcttaatgactttgtctctgatgcagattcaactttgattggtgattgtgcaactgtacatacagctaataaatgggatctcattattagtgatatgtacgaccctaagactaaaaatgttacaaaagaaaatgactctaaagagggttttttcacttacatttgtgggtttatacaacaaaagctagctcttggaggttccgtggctataaagataacagaacattcttggaatgctgatctttataagctcatgggacacttcgcatggtggacagcctttgttactaatgtgaatgcgtcatcatctgaagcatttttaattggatgtaattatcttggcaaaccacgcgaacaaatagatggttatgtcatgcatgcaaattacatattttggaggaatacaaatccaattcagttgtcttcctattctttatttgacatgagtaaatttccccttaaattaaggggtactgctgttatgtctttaaaagaaggtcaaatcaatgatatgattttatctcttcttagtaaaggtagacttataattagagaaaacaacagagttgttatttctagtgatgttcttgttaacaactaaacgaacaatgtttgtttttcttgttttattgccactagtctctagtcagtgtgttaatcttacaaccagaactcaattaccccctgcatacactaattctttcacacgtggtgtttattaccctgacaaagttttcagatcctcagttttacattcaactcaggacttgttcttacctttcttttccaatgttacttggttccatgctatacatgtctctgggaccaatggtactaagaggtttgataaccctgtcctaccatttaatgatggtgtttattttgcttccactgagaagtctaacataataagaggctggatttttggtactactttagactcgaagacccagtccctacttattgttaataacgctactaatgttgttattaaagtctgtgaatttcaattttgtaatgatccatttttgggtgtttattaccacaaaaacaacaaaagttggatggaaagtgagttcagagtttattctagtgcgaataattgcacttttgaatatgtctctcagccttttcttatggaccttgaaggaaaacagggtaatttcaaaaatcttagggaatttgtgtttaagaatattgatggttattttaaaatatattctaagcacacgcctattaatttagtgcgtgatctccctcagggtttttcggctttagaaccattggtagatttgccaataggtattaacatcactaggtttcaaactttacttgctttacatagaagttatttgactcctggtgattcttcttcaggttggacagctggtgctgcagcttattatgtgggttatcttcaacctaggacttttctattaaaatataatgaaaatggaaccattacagatgctgtagactgtgcacttgaccctctctcagaaacaaagtgtacgttgaaatccttcactgtagaaaaaggaatctatcaaacttctaactttagagtccaaccaacagaatctattgttagatttcctaatattacaaacttgtgcccttttggtgaagtttttaacgccaccagatttgcatctgtttatgcttggaacaggaagagaatcagcaactgtgttgctgattattctgtcctatataattccgcatcattttccacttttaagtgttatggagtgtctcctactaaattaaatgatctctgctttactaatgtctatgcagattcatttgtaattagaggtgatgaagtcagacaaatcgctccagggcaaactggaaagattgctgattataattataaattaccagatgattttacaggctgcgttatagcttggaattctaacaatcttgattctaaggttggtggtaattataattacctgtatagattgtttaggaagtctaatctcaaaccttttgagagagatatttcaactgaaatctatcaggccggtagcacaccttgtaatggtgttgaaggttttaattgttactttcctttacaatcatatggtttccaacccactaatggtgttggttaccaaccatacagagtagtagtactttcttttgaacttctacatgcaccagcaactgtttgtggacctaaaaagtctactaatttggttaaaaacaaatgtgtcaatttcaacttcaatggtttaacaggcacaggtgttcttactgagtctaacaaaaagtttctgcctttccaacaatttggcagagacattgctgacactactgatgctgtccgtgatccacagacacttgagattcttgacattacaccatgttcttttggtggtgtcagtgttataacaccaggaacaaatacttctaaccaggttgctgttctttatcaggatgttaactgcacagaagtccctgttgctattcatgcagatcaacttactcctacttggcgtgtttattctacaggttctaatgtttttcaaacacgtgcaggctgtttaataggggctgaacatgtcaacaactcatatgagtgtgacatacccattggtgcaggtatatgcgctagttatcagactcagactaattctcctcggcgggcacgtagtgtagctagtcaatccatcattgcctacactatgtcacttggtgcagaaaattcagttgcttactctaataactctattgccatacccacaaattttactattagtgttaccacagaaattctaccagtgtctatgaccaagacatcagtagattgtacaatgtacatttgtggtgattcaactgaatgcagcaatcttttgttgcaatatggcagtttttgtacacaattaaaccgtgctttaactggaatagctgttgaacaagacaaaaacacccaagaagtttttgcacaagtcaaacaaatttacaaaacaccaccaattaaagattttggtggttttaatttttcacaaatattaccagatccatcaaaaccaagcaagaggtcatttattgaagatctacttttcaacaaagtgacacttgcagatgctggcttcatcaaacaatatggtgattgccttggtgatattgctgctagagacctcatttgtgcacaaaagtttaacggccttactgttttgccacctttgctcacagatgaaatgattgctcaatacacttctgcactgttagcgggtacaatcacttctggttggacctttggtgcaggtgctgcattacaaataccatttgctatgcaaatggcttataggtttaatggtattggagttacacagaatgttctctatgagaaccaaaaattgattgccaaccaatttaatagtgctattggcaaaattcaagactcactttcttccacagcaagtgcacttggaaaacttcaagatgtggtcaaccaaaatgcacaagctttaaacacgcttgttaaacaacttagctccaattttggtgcaatttcaagtgttttaaatgatatcctttcacgtcttgacaaagttgaggctgaagtgcaaattgataggttgatcacaggcagacttcaaagtttgcagacatatgtgactcaacaattaattagagctgcagaaatcagagcttctgctaatcttgctgctactaaaatgtcagagtgtgtacttggacaatcaaaaagagttgatttttgtggaaagggctatcatcttatgtccttccctcagtcagcacctcatggtgtagtcttcttgcatgtgacttatgtccctgcacaagaaaagaacttcacaactgctcctgccatttgtcatgatggaaaagcacactttcctcgtgaaggtgtctttgtttcaaatggcacacactggtttgtaacacaaaggaatttttatgaaccacaaatcattactacagacaacacatttgtgtctggtaactgtgatgttgtaataggaattgtcaacaacacagtttatgatcctttgcaacctgaattagactcattcaaggaggagttagataaatattttaagaatcatacatcaccagatgttgatttaggtgacatctctggcattaatgcttcagttgtaaacattcaaaaagaaattgaccgcctcaatgaggttgccaagaatttaaatgaatctctcatcgatctccaagaacttggaaagtatgagcagtatataaaatggccatggtacatttggctaggttttatagctggcttgattgccatagtaatggtgacaattatgctttgctgtatgaccagttgctgtagttgtctcaagggctgttgttcttgtggatcctgctgcaaatttgatgaagacgactctgagccagtgctcaaaggagtcaaattacattacacataaacgaacttatggatttgtttatgagaatcttcacaattggaactgtaactttgaagcaaggtgaaatcaaggatgctactccttcagattttgttcgcgctactgcaacgataccgatacaagcctcactccctttcggatggcttattgttggcgttgcacttcttgctgtttttcagagcgcttccaaaatcataaccctcaaaaagagatggcaactagcactctccaagggtgttcactttgtttgcaacttgctgttgttgtttgtaacagtttactcacaccttttgctcgttgctgctggccttgaagccccttttctctatctttatgctttagtctacttcttgcagagtataaactttgtaagaataataatgaggctttggctttgctggaaatgccgttccaaaaacccattactttatgatgccaactattttctttgctggcatactaattgttacgactattgtataccttacaatagtgtaacttcttcaattgtcattacttcaggtgatggcacaacaagtcctatttctgaacatgactaccagattggtggttatactgaaaaatgggaatctggagtaaaagactgtgttgtattacacagttacttcacttcagactattaccagctgtactcaactcaattgagtacagacactggtgttgaacatgttaccttcttcatctacaataaaattgttgatgagcctgaagaacatgtccaaattcacacaatcgacggttcatccggagttgttaatccagtaatggaaccaatttatgatgaaccgacgacgactactagcgtgcctttgtaagcacaagctgatgagtacgaacttatgtactcattcgtttcggaagagacaggtacgttaatagttaatagcgtacttctttttcttgctttcgtggtattcttgctagttacactagccatccttactgcgcttcgattgtgtgcgtactgctgcaatattgttaacgtgagtcttgtaaaaccttctttttacgtttactctcgtgttaaaaatctgaattcttctagagttcctgatcttctggtctaaacgaactaaatattatattagtttttctgtttggaactttaattttagccatggcagattccaacggtactattaccgttgaagagcttaaaaagctccttgaacaatggaacctagtaataggtttcctattccttacatggatttgtcttctacaatttgcctatgccaacaggaataggtttttgtatataattaagttaattttcctctggctgttatggccagtaactttagcttgttttgtgcttgctgctgtttacagaataaattggatcaccggtggaattgctatcgcaatggcttgtcttgtaggcttgatgtggctcagctacttcattgcttctttcagactgtttgcgcgtacgcgatccatgtggtcattcaatccagaaactaacattcttctcaacgtgccactccatggcactattctgaccagaccgcttctagaaagtgaactcgtaatcggagctgtgatccttcgtggacatcttcgtattgctggacaccatctaggacgctgtgacatcaaggacctgcctaaagaaatcactgttgctacatcacgaacgctttcttattacaaattgggagcttcgcagcgtgtagcaggtgactcaggttttgctgcatacagtcgctacaggattggcaactataaattaaacacagaccattccagtagcagtgacaatattgctttgcttgtacagtaagtgacaacagatgtttcatctcgttgactttcaggttactatagcagagatattactaattattatgaggacttttaaagtttccatttggaatcttgattacatcataaacctcataattaaaaatttatctaagtcactaactgagaataaatattctcaattagatgaagagcaaccaatggagattgattaaacgaacatgaaaattattcttttcttggcactgataacactcgctacttgtgagctttatcactaccaagagtgtgttagaggtacaacagtacttttaaaagaaccttgctcttctggaacatacgagggcaattcaccatttcatcctctagctgataacaaatttgcactgacttgctttagcactcaatttgcttttgcttgtcctgacggcgtaaaacacgtctatcagttacgtgccagatcagtttcacctaaactgttcatcagacaagaggaagttcaagaactttactctccaatttttcttattgttgcggcaatagtgtttataacactttgcttcacactcaaaagaaagacagaatgattgaactttcattaattgacttctatttgtgctttttagcctttctgctattccttgttttaattatgcttattatcttttggttctcacttgaactgcaagatcataatgaaacttgtcacgcctaaacgaacatgaaatttcttgttttcttaggaatcatcacaactgtagctgcatttcaccaagaatgtagtttacagtcatgtactcaacatcaaccatatgtagttgatgacccgtgtcctattcacttctattctaaatggtatattagagtaggagctagaaaatcagcacctttaattgaattgtgcgtggatgaggctggttctaaatcacccattcagtacatcgatatcggtaattatacagtttcctgttcaccttttacaattaattgccaggaacctaaattgggtagtcttgtagtgcgttgttcgttctatgaagactttttagagtatcatgacgttcgtgttgttttagatttcatctaaacgaacaaactaaaatggtgagcaagggcgaggaggataacatggccatcatcaaggagttcatgcgcttcaaggtgcacatggagggctccgtgaacggccacgagttcgagatcgagggcgagggcgagggccgcccctacgagggcacccagaccgccaagctgaaggtgaccaagggtggccccctgcccttcgcctgggacatcctgtcccctcagttcatgtacggctccaaggcctacgtgaagcaccccgccgacatccccgactacttgaagctgtccttccccgagggcttcaagtgggagcgcgtgatgaacttcgaggacggcggcgtggtgaccgtgacccaggactcctccctgcaggacggcgagttcatctacaaggtgaagctgcgcggcaccaacttcccctccgacggccccgtaatgcagaagaagaccatgggctgggaggcctcctccgagcggatgtaccccgaggacggcgccctgaagggcgagatcaagcagaggctgaagctgaaggacggcggccactacgacgctgaggtcaagaccacctacaaggccaagaagcccgtgcagctgcccggcgcctacaacgtcaacatcaagttggacatcacctcccacaacgaggactacaccatcgtggaacagtacgaacgcgccgagggccgccactccaccggcggcatggacgagctgtacaaagggtccggagccacgaacttctctctgttaaagcaagcaggggacgtggaagaaaaccccggtccttctgataatggaccccaaaatcagcgaaatgcaccccgcattacgtttggtggaccctcagattcaactggcagtaaccagaatggagaacgcagtggggcgcgatcaaaacaacgtcggccccaaggtttacccaataatactgcgtcttggttcaccgctctcactcaacatggcaaggaagaccttaaattccctcgaggacaaggcgttccaattaacaccaatagcagtccagatgaccaaattggctactaccgaagagctaccagacgaattcgtggtggtgacggtaaaatgaaagatctcagtccaagatggtatttctactacctaggaactgggccagaagctggacttccctatggtgctaacaaagacggcatcatatgggttgcaactgagggagccttgaatacaccaaaagatcacattggcacccgcaatcctgctaacaatgctgcaatcgtgctacaacttcctcaaggaacaacattgccaaaaggcttctacgcagaagggagcagaggcggcagtcaagcctcttctcgttcctcatcacgtagtcgcaacagttcaagaaattcaactccaggcagcagtaggggaacttctcctgctagaatggctggcaatggcggtgatgctgctcttgctttgctgctgcttgacagattgaaccagcttgagagcaaaatgtctggtaaaggccaacaacaacaaggccaaactgtcactaagaaatctgctgctgaggcttctaagaagcctcggcaaaaacgtactgccactaaagcatacaatgtaacacaagctttcggcagacgtggtccagaacaaacccaaggaaattttggggaccaggaactaatcagacaaggaactgattacaaacattggccgcaaattgcacaatttgcccccagcgcttcagcgttcttcggaatgtcgcgcattggcatggaagtcacaccttcgggaacgtggttgacctacacaggtgccatcaaattggatgacaaagatccaaatttcaaagatcaagtcattttgctgaataagcatattgacgcatacaaaacattcccaccaacagagcctaaaaaggacaaaaagaagaaggctgatgaaactcaagccttaccgcagagacagaagaaacagcaaactgtgactcttcttcctgctgcagatttggatgatttctccaaacaattgcaacaatccatgagcagtgctgactcaactcaggcctaaactcatgcagaccacacaaggcagatgggctatataaacgttttcgcttttccgtttacgatatatagtctactcttgtgcagaatgaattctcgtaactacatagcacaagtagatgtagttaactttaatctcacatagcaatctttaatcagtgtgtaacattagggaggacttgaaagagccaccacattttcaccgaggccacgcggagtacgatcgagtgtacagtgaacaatgctagggagagctgcctatatggaagagccctaatgtgtaaaattaattttagtagtgctatccccatgtgattttaatagcttcttaggagaatgacaaaaaaaaaaaaaaaaaaaaaaaaaaaaaaaaa

The mCherry sequences are highlighted in red, the P2A sequences are highlighted in yellow, and the N sequences are highlighted in blue.

**Complete sequences of rSARS-CoV-2 mCherry-SN:**

attaaaggtttataccttcccaggtaacaaaccaaccaactttcgatctcttgtagatctgttctctaaacgaactttaaaatctgtgtggctgtcactcggctgcatgcttagtgcactcacgcagtataattaataactaattactgtcgttgacaggacacgagtaactcgtctatcttctgcaggctgcttacggtttcgtccgtgttgcagccgatcatcagcacatctaggtttcgtccgggtgtgaccgaaaggtaagatggagagccttgtccctggtttcaacgagaaaacacacgtccaactcagtttgcctgttttacaggttcgcgacgtgctcgtacgtggctttggagactccgtggaggaggtcttatcagaggcacgtcaacatcttaaagatggcacttgtggcttagtagaagttgaaaaaggcgttttgcctcaacttgaacagccctatgtgttcatcaaacgttcggatgctcgaactgcacctcatggtcatgttatggttgagctggtagcagaactcgaaggcattcagtacggtcgtagtggtgagacacttggtgtccttgtccctcatgtgggcgaaataccagtggcttaccgcaaggttcttcttcgtaagaacggtaataaaggagctggtggccatagttacggcgccgatctaaagtcatttgacttaggcgacgagcttggcactgatccttatgaagattttcaagaaaactggaacactaaacatagcagtggtgttacccgtgaactcatgcgtgagcttaacggaggggcatacactcgctatgtcgataacaacttctgtggccctgatggctaccctcttgagtgcattaaagaccttctagcacgtgctggtaaagcttcatgcactttgtccgaacaactggactttattgacactaagaggggtgtatactgctgccgtgaacatgagcatgaaattgcttggtacacggaacgttctgaaaagagctatgaattgcagacaccttttgaaattaaattggcaaagaaatttgacaccttcaatggggaatgtccaaattttgtatttcccttaaattccataatcaagactattcaaccaagggttgaaaagaaaaagcttgatggctttatgggtagaattcgatctgtctatccagttgcgtcaccaaatgaatgcaaccaaatgtgcctttcaactctcatgaagtgtgatcattgtggtgaaacttcatggcagacgggcgattttgttaaagccacttgcgaattttgtggcactgagaatttgactaaagaaggtgccactacttgtggttacttaccccaaaatgctgttgttaaaatttattgtccagcatgtcacaattcagaagtaggacctgagcatagtcttgccgaataccataatgaatctggcttgaaaaccattcttcgtaagggtggtcgcactattgcctttggaggctgtgtgttctcttatgttggttgccataacaagtgtgcctattgggttccacgtgctagcgctaacataggttgtaaccatacaggtgttgttggagaaggttccgaaggtcttaatgacaaccttcttgaaatactccaaaaagagaaagtcaacatcaatattgttggtgactttaaacttaatgaagagatcgccattattttggcatctttttctgcttccacaagtgcttttgtggaaactgtgaaaggtttggattataaagcattcaaacaaattgttgaatcctgtggtaattttaaagttacaaaaggaaaagctaaaaaaggtgcctggaatattggtgaacagaaatcaatactgagtcctctttatgcatttgcatcagaggctgctcgtgttgtacgatcaattttctcccgcactcttgaaactgctcaaaattctgtgcgtgttttacagaaggccgctataacaatactagatggaatttcacagtattcactgagactcattgatgctatgatgttcacatctgatttggctactaacaatctagttgtaatggcctacattacaggtggtgttgttcagttgacttcgcagtggctaactaacatctttggcactgtttatgaaaaactcaaacccgtccttgattggcttgaagagaagtttaaggaaggtgtagagtttcttagagacggttgggaaattgttaaatttatctcaacctgtgcttgtgaaattgtcggtggacaaattgtcacctgtgcaaaggaaattaaggagagtgttcagacattctttaagcttgtaaataaatttttggctttgtgtgctgactctatcattattggtggagctaaacttaaagccttgaatttaggtgaaacatttgtcacgcactcaaagggattgtacagaaagtgtgttaaatccagagaagaaactggcctactcatgcctctaaaagccccaaaagaaattatcttcttagagggagaaacacttcccacagaagtgttaacagaggaagttgtcttgaaaactggtgatttacaaccattagaacaacctactagtgaagctgttgaagctccattggttggtacaccagtttgtattaacgggcttatgttgctcgaaatcaaagacacagaaaagtactgtgcccttgcacctaatatgatggtaacaaacaataccttcacactcaaaggcggtgcaccaacaaaggttacttttggtgatgacactgtgatagaagtgcaaggttacaagagtgtgaatatcacttttgaacttgatgaaaggattgataaagtacttaatgagaagtgctctgcctatacagttgaactcggtacagaagtaaatgagttcgcctgtgttgtggcagatgctgtcataaaaactttgcaaccagtatctgaattacttacaccactgggcattgatttagatgagtggagtatggctacatactacttatttgatgagtctggtgagtttaaattggcttcacatatgtattgttctttctaccctccagatgaggatgaagaagaaggtgattgtgaagaagaagagtttgagccatcaactcaatatgagtatggtactgaagatgattaccaaggtaaacctttggaatttggtgccacttctgctgctcttcaacctgaagaagagcaagaagaagattggttagatgatgatagtcaacaaactgttggtcaacaagacggcagtgaggacaatcagacaactactattcaaacaattgttgaggttcaacctcaattagagatggaacttacaccagttgttcagactattgaagtgaatagttttagtggttatttaaaacttactgacaatgtatacattaaaaatgcagacattgtggaagaagctaaaaaggtaaaaccaacagtggttgttaatgcagccaatgtttaccttaaacatggaggaggtgttgcaggagccttaaataaggctactaacaatgccatgcaagttgaatctgatgattacatagctactaatggaccacttaaagtgggtggtagttgtgttttaagcggacacaatcttgctaaacactgtcttcatgttgtcggcccaaatgttaacaaaggtgaagacattcaacttcttaagagtgcttatgaaaattttaatcagcacgaagttctacttgcaccattattatcagctggtatttttggtgctgaccctatacattctttaagagtttgtgtagatactgttcgcacaaatgtctacttagctgtctttgataaaaatctctatgacaaacttgtttcaagctttttggaaatgaagagtgaaaagcaagttgaacaaaagatcgctgagattcctaaagaggaagttaagccatttataactgaaagtaaaccttcagttgaacagagaaaacaagatgataagaaaatcaaagcttgtgttgaagaagttacaacaactctggaagaaactaagttcctcacagaaaacttgttactttatattgacattaatggcaatcttcatccagattctgccactcttgttagtgacattgacatcactttcttaaagaaagatgctccatatatagtgggtgatgttgttcaagagggtgttttaactgctgtggttatacctactaaaaaggctggtggcactactgaaatgctagcgaaagctttgagaaaagtgccaacagacaattatataaccacttacccgggtcagggtttaaatggttacactgtagaggaggcaaagacagtgcttaaaaagtgtaaaagtgccttttacattctaccatctattatctctaatgagaagcaagaaattcttggaactgtttcttggaatttgcgagaaatgcttgcacatgcagaagaaacacgcaaattaatgcctgtctgtgtggaaactaaagccatagtttcaactatacagcgtaaatataagggtattaaaatacaagagggtgtggttgattatggtgctagattttacttttacaccagtaaaacaactgtagcgtcacttatcaacacacttaacgatctaaatgaaactcttgttacaatgccacttggctatgtaacacatggcttaaatttggaagaagctgctcggtatatgagatctctcaaagtgccagctacagtttctgtttcttcacctgatgctgttacagcgtataatggttatcttacttcttcttctaaaacacctgaagaacattttattgaaaccatctcacttgctggttcctataaagattggtcctattctggacaatctacacaactaggtatagaatttcttaagagaggtgataaaagtgtatattacactagtaatcctaccacattccacctagatggtgaagttatcacctttgacaatcttaagacacttctttctttgagagaagtgaggactattaaggtgtttacaacagtagacaacattaacctccacacgcaagttgtggacatgtcaatgacatatggacaacagtttggtccaacttatttggatggagctgatgttactaaaataaaacctcataattcacatgaaggtaaaacattttatgttttacctaatgatgacactctacgtgttgaggcttttgagtactaccacacaactgatcctagttttctgggtaggtacatgtcagcattaaatcacactaaaaagtggaaatacccacaagttaatggtttaacttctattaaatgggcagataacaactgttatcttgccactgcattgttaacactccaacaaatagagttgaagtttaatccacctgctctacaagatgcttattacagagcaagggctggtgaagctgctaacttttgtgcacttatcttagcctactgtaataagacagtaggtgagttaggtgatgttagagaaacaatgagttacttgtttcaacatgccaatttagattcttgcaaaagagtcttgaacgtggtgtgtaaaacttgtggacaacagcagacaacccttaagggtgtagaagctgttatgtacatgggcacactttcttatgaacaatttaagaaaggtgttcagataccttgtacgtgtggtaaacaagctacaaaatatctagtacaacaggagtcaccttttgttatgatgtcagcaccacctgctcagtatgaacttaagcatggtacatttacttgtgctagtgagtacactggtaattaccagtgtggtcactataaacatataacttctaaagaaactttgtattgcatagacggtgctttacttacaaagtcctcagaatacaaaggtcctattacggatgttttctacaaagaaaacagttacacaacaaccataaaaccagttacttataaattggatggtgttgtttgtacagaaattgaccctaagttggacaattattataagaaagacaattcttatttcacagagcaaccaattgatcttgtaccaaaccaaccatatccaaacgcaagcttcgataattttaagtttgtatgtgataatatcaaatttgctgatgatttaaaccagttaactggttataagaaacctgcttcaagagagcttaaagttacatttttccctgacttaaatggtgatgtggtggctattgattataaacactacacaccctcttttaagaaaggagctaaattgttacataaacctattgtttggcatgttaacaatgcaactaataaagccacgtataaaccaaatacctggtgtatacgttgtctttggagcacaaaaccagttgaaacatcaaattcgtttgatgtactgaagtcagaggacgcgcagggaatggataatcttgcctgcgaagatctaaaaccagtctctgaagaagtagtggaaaatcctaccatacagaaagacgttcttgagtgtaatgtgaaaactaccgaagttgtaggagacattatacttaaaccagcaaataatagtttaaaaattacagaagaggttggccacacagatctaatggctgcttatgtagacaattctagtcttactattaagaaacctaatgaattatctagagtattaggtttgaaaacccttgctactcatggtttagctgctgttaatagtgtcccttgggatactatagctaattatgctaagccttttcttaacaaagttgttagtacaactactaacatagttacacggtgtttaaaccgtgtttgtactaattatatgccttatttctttactttattgctacaattgtgtacttttactagaagtacaaattctagaattaaagcatctatgccgactactatagcaaagaatactgttaagagtgtcggtaaattttgtctagaggcttcatttaattatttgaagtcacctaatttttctaaactgataaatattataatttggtttttactattaagtgtttgcctaggttctttaatctactcaaccgctgctttaggtgttttaatgtctaatttaggcatgccttcttactgtactggttacagagaaggctatttgaactctactaatgtcactattgcaacctactgtactggttctataccttgtagtgtttgtcttagtggtttagattctttagacacctatccttctttagaaactatacaaattaccatttcatcttttaaatgggatttaactgcttttggcttagttgcagagtggtttttggcatatattcttttcactaggtttttctatgtacttggattggctgcaatcatgcaattgtttttcagctattttgcagtacattttattagtaattcttggcttatgtggttaataattaatcttgtacaaatggccccgatttcagctatggttagaatgtacatcttctttgcatcattttattatgtatggaaaagttatgtgcatgttgtagacggttgtaattcatcaacttgtatgatgtgttacaaacgtaatagagcaacaagagtcgaatgtacaactattgttaatggtgttagaaggtccttttatgtctatgctaatggaggtaaaggcttttgcaaactacacaattggaattgtgttaattgtgatacattctgtgctggtagtacatttattagtgatgaagttgcgagagacttgtcactacagtttaaaagaccaataaatcctactgaccagtcttcttacatcgttgatagtgttacagtgaagaatggttccatccatctttactttgataaagctggtcaaaagacttatgaaagacattctctctctcattttgttaacttagacaacctgagagctaataacactaaaggttcattgcctattaatgttatagtttttgatggtaaatcaaaatgtgaagaatcatctgcaaaatcagcgtctgtttactacagtcagcttatgtgtcaacctatactgttactagatcaggcattagtgtctgatgttggtgatagtgcggaagttgcagttaaaatgtttgatgcttacgttaatacgttttcatcaacttttaacgtaccaatggaaaaactcaaaacactagttgcaactgcagaagctgaacttgcaaagaatgtgtccttagacaatgtcttatctacttttatttcagcagctcggcaagggtttgttgattcagatgtagaaactaaagatgttgttgaatgtcttaaattgtcacatcaatctgacatagaagttactggcgatagttgtaataactatatgctcacctataacaaagttgaaaacatgacaccccgtgaccttggtgcttgtattgactgtagtgcgcgtcatattaatgcgcaggtagcaaaaagtcacaacattgctttgatatggaacgttaaagatttcatgtcattgtctgaacaactacgaaaacaaatacgtagtgctgctaaaaagaataacttaccttttaagttgacatgtgcaactactagacaagttgttaatgttgtaacaacaaagatagcacttaagggtggtaaaattgttaataattggttgaagcagttaattaaagttacacttgtgttcctttttgttgctgctattttctatttaataacacctgttcatgtcatgtctaaacatactgacttttcaagtgaaatcataggatacaaggctattgatggtggtgtcactcgtgacatagcatctacagatacttgttttgctaacaaacatgctgattttgacacatggtttagtcagcgtggtggtagttatactaatgacaaagcttgcccattgattgctgcagtcataacaagagaagtgggttttgtcgtgcctggtttgcctggcacgatattacgcacaactaatggtgactttttgcatttcttacctagagtttttagtgcagttggtaacatctgttacacaccatcaaaacttatagagtacactgactttgcaacatcagcttgtgttttggctgctgaatgtacaatttttaaagatgcttctggtaagccagtaccatattgttatgataccaatgtactagaaggttctgttgcttatgaaagtttacgccctgacacacgttatgtgctcatggatggctctattattcaatttcctaacacctaccttgaaggttctgttagagtggtaacaacttttgattctgagtactgtaggcacggcacttgtgaaagatcagaagctggtgtttgtgtatctactagtggtagatgggtacttaacaatgattattacagatctttaccaggagttttctgtggtgtagatgctgtaaatttacttactaatatgtttacaccactaattcaacctattggtgctttggacatatcagcatctatagtagctggtggtattgtagctatcgtagtaacatgccttgcctactattttatgaggtttagaagagcttttggtgaatacagtcatgtagttgcctttaatactttactattccttatgtcattcactgtactctgtttaacaccagtttactcattcttacctggtgtttattctgttatttacttgtacttgacattttatcttactaatgatgtttcttttttagcacatattcagtggatggttatgttcacacctttagtacctttctggataacaattgcttatatcatttgtatttccacaaagcatttctattggttctttagtaattacctaaagagacgtgtagtctttaatggtgtttcctttagtacttttgaagaagctgcgctgtgcacctttttgttaaataaagaaatgtatctaaagttgcgtagtgatgtgctattacctcttacgcaatataatagatacttagctctttataataagtacaagtattttagtggagcaatggatacaactagctacagagaagctgcttgttgtcatctcgcaaaggctctcaatgacttcagtaactcaggttctgatgttctttaccaaccaccacaaacctctatcacctcagctgttttgcagagtggttttagaaaaatggcattcccatctggtaaagttgagggttgtatggtacaagtaacttgtggtacaactacacttaacggtctttggcttgatgacgtagtttactgtccaagacatgtgatctgcacctctgaagacatgcttaaccctaattatgaagatttactcattcgtaagtctaatcataatttcttggtacaggctggtaatgttcaactcagggttattggacattctatgcaaaattgtgtacttaagcttaaggttgatacagccaatcctaagacacctaagtataagtttgttcgcattcaaccaggacagactttttcagtgttagcttgttacaatggttcaccatctggtgtttaccaatgtgctatgaggcccaatttcactattaagggttcattccttaatggttcatgtggtagtgttggttttaacatagattatgactgtgtctctttttgttacatgcaccatatggaattaccaactggagttcatgctggcacagacttagaaggtaacttttatggaccttttgttgacaggcaaacagcacaagcagctggtacggacacaactattacagttaatgttttagcttggttgtacgctgctgttataaatggagacaggtggtttctcaatcgatttaccacaactcttaatgactttaaccttgtggctatgaagtacaattatgaacctctaacacaagaccatgttgacatactaggacctctttctgctcaaactggaattgccgttttagatatgtgtgcttcattaaaagaattactgcaaaatggtatgaatggacgtaccatattgggtagtgctttattagaagatgaatttacaccttttgatgttgttagacaatgctcaggtgttactttccaaagtgcagtgaaaagaacaatcaagggtacacaccactggttgttactcacaattttgacttcacttttagttttagtccagagtactcaatggtctttgttcttttttttgtatgaaaatgcctttttaccttttgctatgggtattattgctatgtctgcttttgcaatgatgtttgtcaaacataagcatgcatttctctgtttgtttttgttaccttctcttgccactgtagcttattttaatatggtctatatgcctgctagttgggtgatgcgtattatgacatggttggatatggttgatactagtttgtctggttttaagctaaaagactgtgttatgtatgcatcagctgtagtgttactaatccttatgacagcaagaactgtgtatgatgatggtgctaggagagtgtggacacttatgaatgtcttgacactcgtttataaagtttattatggtaatgctttagatcaagccatttccatgtgggctcttataatctctgttacttctaactactcaggtgtagttacaactgtcatgtttttggccagaggtattgtttttatgtgtgttgagtattgccctattttcttcataactggtaatacacttcagtgtataatgctagtttattgtttcttaggctatttttgtacttgttactttggcctcttttgtttactcaaccgctactttagactgactcttggtgtttatgattacttagtttctacacaggagtttagatatatgaattcacagggactactcccacccaagaatagcatagatgccttcaaactcaacattaaattgttgggtgttggtggcaaaccttgtatcaaagtagccactgtacagtctaaaatgtcagatgtaaagtgcacatcagtagtcttactctcagttttgcaacaactcagagtagaatcatcatctaaattgtgggctcaatgtgtccagttacacaatgacattctcttagctaaagatactactgaagcctttgaaaaaatggtttcactactttctgttttgctttccatgcagggtgctgtagacataaacaagctttgtgaagaaatgctggacaacagggcaaccttacaagctatagcctcagagtttagttcccttccatcatatgcagcttttgctactgctcaagaagcttatgagcaggctgttgctaatggtgattctgaagttgttcttaaaaagttgaagaagtctttgaatgtggctaaatctgaatttgaccgtgatgcagccatgcaacgtaagttggaaaagatggctgatcaagctatgacccaaatgtataaacaggctagatctgaggacaagagggcaaaagttactagtgctatgcagacaatgcttttcactatgcttagaaagttggataatgatgcactcaacaacattatcaacaatgcaagagatggttgtgttcccttgaacataatacctcttacaacagcagccaaactaatggttgtcataccagactataacacatataaaaatacgtgtgatggtacaacatttacttatgcatcagcattgtgggaaatccaacaggttgtagatgcagatagtaaaattgttcaacttagtgaaattagtatggacaattcacctaatttagcatggcctcttattgtaacagctttaagggccaattctgctgtcaaattacagaataatgagcttagtcctgttgcactacgacagatgtcttgtgctgccggtactacacaaactgcttgcactgatgacaatgcgttagcttactacaacacaacaaagggaggtaggtttgtacttgcactgttatccgatttacaggatttgaaatgggctagattccctaagagtgatggaactggtactatctatacagaactggaaccaccttgtaggtttgttacagacacacctaaaggtcctaaagtgaagtatttatactttattaaaggattaaacaacctaaatagaggtatggtacttggtagtttagctgccacagtacgtctacaagctggtaatgcaacagaagtgcctgccaattcaactgtattatctttctgtgcttttgctgtagatgctgctaaagcttacaaagattatctagctagtgggggacaaccaatcactaattgtgttaagatgttgtgtacacacactggtactggtcaggcaataacagttacaccggaagccaatatggatcaagaatcctttggtggtgcatcgtgttgtctgtactgccgttgccacatagatcatccaaatcctaaaggattttgtgacttaaaaggtaagtatgtacaaatacctacaacttgtgctaatgaccctgtgggttttacacttaaaaacacagtctgtaccgtctgcggtatgtggaaaggttatggctgtagttgtgatcaactccgcgaacccatgcttcagtcagctgatgcacaatcgtttttaaacgggtttgcggtgtaagtgcagcccgtcttacaccgtgcggcacaggcactagtactgatgtcgtatacagggcttttgacatctacaatgataaagtagctggttttgctaaattcctaaaaactaattgttgtcgcttccaagaaaaggacgaagatgacaatttaattgattcttactttgtagttaagagacacactttctctaactaccaacatgaagaaacaatttataatttacttaaggattgtccagctgttgctaaacatgacttctttaagtttagaatagacggtgacatggtaccacatatatcacgtcaacgtcttactaaatacacaatggcagacctcgtctatgctttaaggcattttgatgaaggtaattgtgacacattaaaagaaatacttgtcacatacaattgttgtgatgatgattatttcaataaaaaggactggtatgattttgtagaaaacccagatatattacgcgtatacgccaacttaggtgaacgtgtacgccaagctttgttaaaaacagtacaattctgtgatgccatgcgaaatgctggtattgttggtgtactgacattagataatcaagatctcaatggtaactggtatgatttcggtgatttcatacaaaccacgccaggtagtggagttcctgttgtagattcttattattcattgttaatgcctatattaaccttgaccagggctttaactgcagagtcacatgttgacactgacttaacaaagccttacattaagtgggatttgttaaaatatgacttcacggaagagaggttaaaactctttgaccgttattttaaatattgggatcagacataccacccaaattgtgttaactgtttggatgacagatgcattctgcattgtgcaaactttaatgttttattctctacagtgttcccacctacaagttttggaccactagtgagaaaaatatttgttgatggtgttccatttgtagtttcaactggataccacttcagagagctaggtgttgtacataatcaggatgtaaacttacatagctctagacttagttttaaggaattacttgtgtatgctgctgaccctgctatgcacgctgcttctggtaatctattactagataaacgcactacgtgcttttcagtagctgcacttactaacaatgttgcttttcaaactgtcaaacccggtaattttaacaaagacttctatgactttgctgtgtctaagggtttctttaaggaaggaagttctgttgaattaaaacacttcttctttgctcaggatggtaatgctgctatcagcgattatgactactatcgttataatctaccaacaatgtgtgatatcagacaactactatttgtagttgaagttgttgataagtactttgattgttacgatggtggctgtattaatgctaaccaagtcatcgtcaacaacctagacaaatcagctggttttccatttaataaatggggtaaggctagactttattatgattcaatgagttatgaggatcaagatgcacttttcgcatatacaaaacgtaatgtcatccctactataactcaaatgaatcttaagtatgccattagtgcaaagaatagagctcgcaccgtagctggtgtctctatctgtagtactatgaccaatagacagtttcatcaaaaattattgaaatcaatagccgccactagaggagctactgtagtaattggaacaagcaaattctatggtggttggcacaacatgttaaaaactgtttatagtgatgtagaaaaccctcaccttatgggttgggattatcctaaatgtgatagagccatgcctaacatgcttagaattatggcctcacttgttcttgctcgcaaacatacaacgtgttgtagcttgtcacaccgtttctatagattagctaatgagtgtgctcaagtattgagtgaaatggtcatgtgtggcggttcactatatgttaaaccaggtggaacctcatcaggagatgccacaactgcttatgctaatagtgtttttaacatttgtcaagctgtcacggccaatgttaatgcacttttatctactgatggtaacaaaattgccgataagtatgtccgcaatttacaacacagactttatgagtgtctctatagaaatagagatgttgacacagactttgtgaatgagttttacgcatatttgcgtaaacatttctcaatgatgatactctctgacgatgctgttgtgtgtttcaatagcacttatgcatctcaaggtctagtggctagcataaagaactttaagtcagttctttattatcaaaacaatgtttttatgtctgaagcaaaatgttggactgagactgaccttactaaaggacctcatgaattttgctctcaacatacaatgctagttaaacagggtgatgattatgtgtaccttccttacccagatccatcaagaatcctaggggccggctgttttgtagatgatatcgtaaaaacagatggtacacttatgattgaacggttcgtgtctttagctatagatgcttacccacttactaaacatcctaatcaggagtatgctgatgtctttcatttgtacttacaatacataagaaagctacatgatgagttaacaggacacatgttagacatgtattctgttatgcttactaatgataacacttcaaggtattgggaacctgagttttatgaggctatgtacacaccgcatacagtcttacaggctgttggggcttgtgttctttgcaattcacagacttcattaagatgtggtgcttgcatacgtagaccattcttatgttgtaaatgctgttacgaccatgtcatatcaacatcacataaattagtcttgtctgttaatccgtatgtttgcaatgctccaggttgtgatgtcacagatgtgactcaactttacttaggaggtatgagctattattgtaaatcacataaaccacccattagttttccattgtgtgctaatggacaagtttttggtttatataaaaatacatgtgttggtagcgataatgttactgactttaatgcaattgcaacatgtgactggacaaatgctggtgattacattttagctaacacctgtactgaaagactcaagctttttgcagcagaaacgctcaaagctactgaggagacatttaaactgtcttatggtattgctactgtacgtgaagtgctgtctgacagagaattacatctttcatgggaagttggtaaacctagaccaccacttaaccgaaattatgtctttactggttatcgtgtaactaaaaacagtaaagtacaaataggagagtacacctttgaaaaaggtgactatggtgatgctgttgtttaccgaggtacaacaacttacaaattaaatgttggtgattattttgtgctgacatcacatacagtaatgccattaagtgcacctacactagtgccacaagagcactatgttagaattactggcttatacccaacactcaatatctcagatgagttttctagcaatgttgcaaattatcaaaaggttggtatgcaaaagtattctacactccagggaccacctggtactggtaagagtcattttgctattggcctagctctctactacccttctgctcgcatagtgtatacagcttgctctcatgccgctgttgatgcactatgtgagaaggcattaaaatatttgcctatagataaatgtagtagaattatacctgcacgtgctcgtgtagagtgttttgataaattcaaagtgaattcaacattagaacagtatgtcttttgtactgtaaatgcattgcctgagacgacagcagatatagttgtctttgatgaaatttcaatggccacaaattatgatttgagtgttgtcaatgccagattacgtgctaagcactatgtgtacattggcgaccctgctcaattacctgcaccacgcacattgctaactaagggcacactagaaccagaatatttcaattcagtgtgtagacttatgaaaactataggtccagacatgttcctcggaacttgtcggcgttgtcctgctgaaattgttgacactgtgagtgctttggtttatgataataagcttaaagcacataaagacaaatcagctcaatgctttaaaatgttttataagggtgttatcacgcatgatgtttcatctgcaattaacaggccacaaataggcgtggtaagagaattccttacacgtaaccctgcttggagaaaagctgtctttatttcaccttataattcacagaatgctgtagcctcaaagattttgggactaccaactcaaactgttgattcatcacagggctcagaatatgactatgtcatattcactcaaaccactgaaacagctcactcttgtaatgtaaacagatttaatgttgctattaccagagcaaaagtaggcatactttgcataatgtctgatagagacctttatgacaagttgcaatttacaagtcttgaaattccacgtaggaatgtggcaactttacaagctgaaaatgtaacaggactttttaaagattgtagtaaggtaatcactgggttacatcctacacaggcacctacacacctcagtgttgacactaaattcaaaactgaaggtttatgtgttgacatacctggcatacctaaggacatgacctatagaagactcatctctatgatgggttttaaaatgaattatcaagttaatggttaccctaacatgtttatcacccgcgaagaagctataagacatgtacgtgcatggattggcttcgatgtcgaggggtgtcatgctactagagaagctgttggtaccaatttacctttacagctaggtttttctacaggtgttaacctagttgctgtacctacaggttatgttgatacacctaataatacagatttttccagagttagtgctaaaccaccgcctggagatcaatttaaacacctcataccacttatgtacaaaggacttccttggaatgtagtgcgtataaagattgtacaaatgttaagtgacacacttaaaaatctctctgacagagtcgtatttgtcttatgggcacatggctttgagttgacatctatgaagtattttgtgaaaataggacctgagcgcacctgttgtctatgtgatagacgtgccacatgcttttccactgcttcagacacttatgcctgttggcatcattctattggatttgattacgtctataatccgtttatgattgatgttcaacaatggggttttacaggtaacctacaaagcaaccatgatctgtattgtcaagtccatggtaatgcacatgtagctagttgtgatgcaatcatgactaggtgtctagctgtccacgagtgctttgttaagcgtgttgactggactattgaatatcctataattggtgatgaactgaagattaatgcggcttgtagaaaggttcaacacatggttgttaaagctgcattattagcagacaaattcccagttcttcacgacattggtaaccctaaagctattaagtgtgtacctcaagctgatgtagaatggaagttctatgatgcacagccttgtagtgacaaagcttataaaatagaagaattattctattcttatgccacacattctgacaaattcacagatggtgtatgcctattttggaattgcaatgtcgatagatatcctgctaattccattgtttgtagatttgacactagagtgctatctaaccttaacttgcctggttgtgatggtggcagtttgtatgtaaataaacatgcattccacacaccagcttttgataaaagtgcttttgttaatttaaaacaattaccatttttctattactctgacagtccatgtgagtctcatggaaaacaagtagtgtcagatatagattatgtaccactaaagtctgctacgtgtataacacgttgcaatttaggtggtgctgtctgtagacatcatgctaatgagtacagattgtatctcgatgcttataacatgatgatctcagctggctttagcttgtgggtttacaaacaatttgatacttataacctctggaacacttttacaagacttcagagtttagaaaatgtggcttttaatgttgtaaataagggacactttgatggacaacagggtgaagtaccagtttctatcattaataacactgtttacacaaaagttgatggtgttgatgtagaattgtttgaaaataaaacaacattacctgttaatgtagcatttgagctttgggctaagcgcaacattaaaccagtaccagaggtgaaaatactcaataatttgggtgtggacattgctgctaatactgtgatctgggactacaaaagagatgctccagcacatatatctactattggtgtttgttctatgactgacatagccaagaaaccaactgaaacgatttgtgcaccactcactgtcttttttgatggtagagttgatggtcaagtagacttatttagaaatgcccgtaatggtgttcttattacagaaggtagtgttaaaggtttacaaccatctgtaggtcccaaacaagctagtcttaatggagtcacattaattggagaagccgtaaaaacacagttcaattattataagaaagttgatggtgttgtccaacaattacctgaaacttactttactcagagtagaaatttacaagaatttaaacccaggagtcaaatggaaattgatttcttagaattagctatggatgaattcattgaacggtataaattagaaggctatgccttcgaacatatcgtttatggagattttagtcatagtcagttaggtggtttacatctactgattggactagctaaacgttttaaggaatcaccttttgaattagaagattttattcctatggacagtacagttaaaaactatttcataacagatgcgcaaacaggttcatctaagtgtgtgtgttctgttattgatttattacttgatgattttgttgaaataataaaatcccaagatttatctgtagtttctaaggttgtcaaagtgactattgactatacagaaatttcatttatgctttggtgtaaagatggccatgtagaaacattttacccaaaattacaatctagtcaagcgtggcaaccgggtgttgctatgcctaatctttacaaaatgcaaagaatgctattagaaaagtgtgaccttcaaaattatggtgatagtgcaacattacctaaaggcataatgatgaatgtcgcaaaatatactcaactgtgtcaatatttaaacacattaacattagctgtaccctataatatgagagttatacattttggtgctggttctgataaaggagttgcaccaggtacagctgttttaagacagtggttgcctacgggtacgctgcttgtcgattcagatcttaatgactttgtctctgatgcagattcaactttgattggtgattgtgcaactgtacatacagctaataaatgggatctcattattagtgatatgtacgaccctaagactaaaaatgttacaaaagaaaatgactctaaagagggttttttcacttacatttgtgggtttatacaacaaaagctagctcttggaggttccgtggctataaagataacagaacattcttggaatgctgatctttataagctcatgggacacttcgcatggtggacagcctttgttactaatgtgaatgcgtcatcatctgaagcatttttaattggatgtaattatcttggcaaaccacgcgaacaaatagatggttatgtcatgcatgcaaattacatattttggaggaatacaaatccaattcagttgtcttcctattctttatttgacatgagtaaatttccccttaaattaaggggtactgctgttatgtctttaaaagaaggtcaaatcaatgatatgattttatctcttcttagtaaaggtagacttataattagagaaaacaacagagttgttatttctagtgatgttcttgttaacaactaaacgaacaatgtttgtttttcttgttttattgccactagtctctagtcagtgtgttaatcttacaaccagaactcaattaccccctgcatacactaattctttcacacgtggtgtttattaccctgacaaagttttcagatcctcagttttacattcaactcaggacttgttcttacctttcttttccaatgttacttggttccatgctatacatgtctctgggaccaatggtactaagaggtttgataaccctgtcctaccatttaatgatggtgtttattttgcttccactgagaagtctaacataataagaggctggatttttggtactactttagactcgaagacccagtccctacttattgttaataacgctactaatgttgttattaaagtctgtgaatttcaattttgtaatgatccatttttgggtgtttattaccacaaaaacaacaaaagttggatggaaagtgagttcagagtttattctagtgcgaataattgcacttttgaatatgtctctcagccttttcttatggaccttgaaggaaaacagggtaatttcaaaaatcttagggaatttgtgtttaagaatattgatggttattttaaaatatattctaagcacacgcctattaatttagtgcgtgatctccctcagggtttttcggctttagaaccattggtagatttgccaataggtattaacatcactaggtttcaaactttacttgctttacatagaagttatttgactcctggtgattcttcttcaggttggacagctggtgctgcagcttattatgtgggttatcttcaacctaggacttttctattaaaatataatgaaaatggaaccattacagatgctgtagactgtgcacttgaccctctctcagaaacaaagtgtacgttgaaatccttcactgtagaaaaaggaatctatcaaacttctaactttagagtccaaccaacagaatctattgttagatttcctaatattacaaacttgtgcccttttggtgaagtttttaacgccaccagatttgcatctgtttatgcttggaacaggaagagaatcagcaactgtgttgctgattattctgtcctatataattccgcatcattttccacttttaagtgttatggagtgtctcctactaaattaaatgatctctgctttactaatgtctatgcagattcatttgtaattagaggtgatgaagtcagacaaatcgctccagggcaaactggaaagattgctgattataattataaattaccagatgattttacaggctgcgttatagcttggaattctaacaatcttgattctaaggttggtggtaattataattacctgtatagattgtttaggaagtctaatctcaaaccttttgagagagatatttcaactgaaatctatcaggccggtagcacaccttgtaatggtgttgaaggttttaattgttactttcctttacaatcatatggtttccaacccactaatggtgttggttaccaaccatacagagtagtagtactttcttttgaacttctacatgcaccagcaactgtttgtggacctaaaaagtctactaatttggttaaaaacaaatgtgtcaatttcaacttcaatggtttaacaggcacaggtgttcttactgagtctaacaaaaagtttctgcctttccaacaatttggcagagacattgctgacactactgatgctgtccgtgatccacagacacttgagattcttgacattacaccatgttcttttggtggtgtcagtgttataacaccaggaacaaatacttctaaccaggttgctgttctttatcaggatgttaactgcacagaagtccctgttgctattcatgcagatcaacttactcctacttggcgtgtttattctacaggttctaatgtttttcaaacacgtgcaggctgtttaataggggctgaacatgtcaacaactcatatgagtgtgacatacccattggtgcaggtatatgcgctagttatcagactcagactaattctcctcggcgggcacgtagtgtagctagtcaatccatcattgcctacactatgtcacttggtgcagaaaattcagttgcttactctaataactctattgccatacccacaaattttactattagtgttaccacagaaattctaccagtgtctatgaccaagacatcagtagattgtacaatgtacatttgtggtgattcaactgaatgcagcaatcttttgttgcaatatggcagtttttgtacacaattaaaccgtgctttaactggaatagctgttgaacaagacaaaaacacccaagaagtttttgcacaagtcaaacaaatttacaaaacaccaccaattaaagattttggtggttttaatttttcacaaatattaccagatccatcaaaaccaagcaagaggtcatttattgaagatctacttttcaacaaagtgacacttgcagatgctggcttcatcaaacaatatggtgattgccttggtgatattgctgctagagacctcatttgtgcacaaaagtttaacggccttactgttttgccacctttgctcacagatgaaatgattgctcaatacacttctgcactgttagcgggtacaatcacttctggttggacctttggtgcaggtgctgcattacaaataccatttgctatgcaaatggcttataggtttaatggtattggagttacacagaatgttctctatgagaaccaaaaattgattgccaaccaatttaatagtgctattggcaaaattcaagactcactttcttccacagcaagtgcacttggaaaacttcaagatgtggtcaaccaaaatgcacaagctttaaacacgcttgttaaacaacttagctccaattttggtgcaatttcaagtgttttaaatgatatcctttcacgtcttgacaaagttgaggctgaagtgcaaattgataggttgatcacaggcagacttcaaagtttgcagacatatgtgactcaacaattaattagagctgcagaaatcagagcttctgctaatcttgctgctactaaaatgtcagagtgtgtacttggacaatcaaaaagagttgatttttgtggaaagggctatcatcttatgtccttccctcagtcagcacctcatggtgtagtcttcttgcatgtgacttatgtccctgcacaagaaaagaacttcacaactgctcctgccatttgtcatgatggaaaagcacactttcctcgtgaaggtgtctttgtttcaaatggcacacactggtttgtaacacaaaggaatttttatgaaccacaaatcattactacagacaacacatttgtgtctggtaactgtgatgttgtaataggaattgtcaacaacacagtttatgatcctttgcaacctgaattagactcattcaaggaggagttagataaatattttaagaatcatacatcaccagatgttgatttaggtgacatctctggcattaatgcttcagttgtaaacattcaaaaagaaattgaccgcctcaatgaggttgccaagaatttaaatgaatctctcatcgatctccaagaacttggaaagtatgagcagtatataaaatggccatggtacatttggctaggttttatagctggcttgattgccatagtaatggtgacaattatgctttgctgtatgaccagttgctgtagttgtctcaagggctgttgttcttgtggatcctgctgcaaatttgatgaagacgactctgagccagtgctcaaaggagtcaaattacattacacataaacgaacttatggatttgtttatgagaatcttcacaattggaactgtaactttgaagcaaggtgaaatcaaggatgctactccttcagattttgttcgcgctactgcaacgataccgatacaagcctcactccctttcggatggcttattgttggcgttgcacttcttgctgtttttcagagcgcttccaaaatcataaccctcaaaaagagatggcaactagcactctccaagggtgttcactttgtttgcaacttgctgttgttgtttgtaacagtttactcacaccttttgctcgttgctgctggccttgaagccccttttctctatctttatgctttagtctacttcttgcagagtataaactttgtaagaataataatgaggctttggctttgctggaaatgccgttccaaaaacccattactttatgatgccaactattttctttgctggcatactaattgttacgactattgtataccttacaatagtgtaacttcttcaattgtcattacttcaggtgatggcacaacaagtcctatttctgaacatgactaccagattggtggttatactgaaaaatgggaatctggagtaaaagactgtgttgtattacacagttacttcacttcagactattaccagctgtactcaactcaattgagtacagacactggtgttgaacatgttaccttcttcatctacaataaaattgttgatgagcctgaagaacatgtccaaattcacacaatcgacggttcatccggagttgttaatccagtaatggaaccaatttatgatgaaccgacgacgactactagcgtgcctttgtaagcacaagctgatgagtacgaacttatgtactcattcgtttcggaagagacaggtacgttaatagttaatagcgtacttctttttcttgctttcgtggtattcttgctagttacactagccatccttactgcgcttcgattgtgtgcgtactgctgcaatattgttaacgtgagtcttgtaaaaccttctttttacgtttactctcgtgttaaaaatctgaattcttctagagttcctgatcttctggtctaaacgaactaaatattatattagtttttctgtttggaactttaattttagccatggcagattccaacggtactattaccgttgaagagcttaaaaagctccttgaacaatggaacctagtaataggtttcctattccttacatggatttgtcttctacaatttgcctatgccaacaggaataggtttttgtatataattaagttaattttcctctggctgttatggccagtaactttagcttgttttgtgcttgctgctgtttacagaataaattggatcaccggtggaattgctatcgcaatggcttgtcttgtaggcttgatgtggctcagctacttcattgcttctttcagactgtttgcgcgtacgcgatccatgtggtcattcaatccagaaactaacattcttctcaacgtgccactccatggcactattctgaccagaccgcttctagaaagtgaactcgtaatcggagctgtgatccttcgtggacatcttcgtattgctggacaccatctaggacgctgtgacatcaaggacctgcctaaagaaatcactgttgctacatcacgaacgctttcttattacaaattgggagcttcgcagcgtgtagcaggtgactcaggttttgctgcatacagtcgctacaggattggcaactataaattaaacacagaccattccagtagcagtgacaatattgctttgcttgtacagtaagtgacaacagatgtttcatctcgttgactttcaggttactatagcagagatattactaattattatgaggacttttaaagtttccatttggaatcttgattacatcataaacctcataattaaaaatttatctaagtcactaactgagaataaatattctcaattagatgaagagcaaccaatggagattgattaaacgaacatgaaaattattcttttcttggcactgataacactcgctacttgtgagctttatcactaccaagagtgtgttagaggtacaacagtacttttaaaagaaccttgctcttctggaacatacgagggcaattcaccatttcatcctctagctgataacaaatttgcactgacttgctttagcactcaatttgcttttgcttgtcctgacggcgtaaaacacgtctatcagttacgtgccagatcagtttcacctaaactgttcatcagacaagaggaagttcaagaactttactctccaatttttcttattgttgcggcaatagtgtttataacactttgcttcacactcaaaagaaagacagaatgattgaactttcattaattgacttctatttgtgctttttagcctttctgctattccttgttttaattatgcttattatcttttggttctcacttgaactgcaagatcataatgaaacttgtcacgcctaaacgaacatgaaatttcttgttttcttaggaatcatcacaactgtagctgcatttcaccaagaatgtagtttacagtcatgtactcaacatcaaccatatgtagttgatgacccgtgtcctattcacttctattctaaatggtatattagagtaggagctagaaaatcagcacctttaattgaattgtgcgtggatgaggctggttctaaatcacccattcagtacatcgatatcggtaattatacagtttcctgttcaccttttacaattaattgccaggaacctaaattgggtagtcttgtagtgcgttgttcgttctatgaagactttttagagtatcatgacgttcgtgttgttttagatttcatctaaacgaacaaactaaaatggtgagcaagggcgaggaggataacatggccatcatcaaggagttcatgcgcttcaaggtgcacatggagggctccgtgaacggccacgagttcgagatcgagggcgagggcgagggccgcccctacgagggcacccagaccgccaagctgaaggtgaccaagggtggccccctgcccttcgcctgggacatcctgtcccctcagttcatgtacggctccaaggcctacgtgaagcaccccgccgacatccccgactacttgaagctgtccttccccgagggcttcaagtgggagcgcgtgatgaacttcgaggacggcggcgtggtgaccgtgacccaggactcctccctgcaggacggcgagttcatctacaaggtgaagctgcgcggcaccaacttcccctccgacggccccgtaatgcagaagaagaccatgggctgggaggcctcctccgagcggatgtaccccgaggacggcgccctgaagggcgagatcaagcagaggctgaagctgaaggacggcggccactacgacgctgaggtcaagaccacctacaaggccaagaagcccgtgcagctgcccggcgcctacaacgtcaacatcaagttggacatcacctcccacaacgaggactacaccatcgtggaacagtacgaacgcgccgagggccgccactccaccggcggcatggacgagctgtacaaagggtccggagccacgaacttctctctgttaaagcaagcaggggacgtggaagaaaaccccggtccttggtcccacccccagttcgagaagagcggctctgataatggaccccaaaatcagcgaaatgcaccccgcattacgtttggtggaccctcagattcaactggcagtaaccagaatggagaacgcagtggggcgcgatcaaaacaacgtcggccccaaggtttacccaataatactgcgtcttggttcaccgctctcactcaacatggcaaggaagaccttaaattccctcgaggacaaggcgttccaattaacaccaatagcagtccagatgaccaaattggctactaccgaagagctaccagacgaattcgtggtggtgacggtaaaatgaaagatctcagtccaagatggtatttctactacctaggaactgggccagaagctggacttccctatggtgctaacaaagacggcatcatatgggttgcaactgagggagccttgaatacaccaaaagatcacattggcacccgcaatcctgctaacaatgctgcaatcgtgctacaacttcctcaaggaacaacattgccaaaaggcttctacgcagaagggagcagaggcggcagtcaagcctcttctcgttcctcatcacgtagtcgcaacagttcaagaaattcaactccaggcagcagtaggggaacttctcctgctagaatggctggcaatggcggtgatgctgctcttgctttgctgctgcttgacagattgaaccagcttgagagcaaaatgtctggtaaaggccaacaacaacaaggccaaactgtcactaagaaatctgctgctgaggcttctaagaagcctcggcaaaaacgtactgccactaaagcatacaatgtaacacaagctttcggcagacgtggtccagaacaaacccaaggaaattttggggaccaggaactaatcagacaaggaactgattacaaacattggccgcaaattgcacaatttgcccccagcgcttcagcgttcttcggaatgtcgcgcattggcatggaagtcacaccttcgggaacgtggttgacctacacaggtgccatcaaattggatgacaaagatccaaatttcaaagatcaagtcattttgctgaataagcatattgacgcatacaaaacattcccaccaacagagcctaaaaaggacaaaaagaagaaggctgatgaaactcaagccttaccgcagagacagaagaaacagcaaactgtgactcttcttcctgctgcagatttggatgatttctccaaacaattgcaacaatccatgagcagtgctgactcaactcaggcctaaactcatgcagaccacacaaggcagatgggctatataaacgttttcgcttttccgtttacgatatatagtctactcttgtgcagaatgaattctcgtaactacatagcacaagtagatgtagttaactttaatctcacatagcaatctttaatcagtgtgtaacattagggaggacttgaaagagccaccacattttcaccgaggccacgcggagtacgatcgagtgtacagtgaacaatgctagggagagctgcctatatggaagagccctaatgtgtaaaattaattttagtagtgctatccccatgtgattttaatagcttcttaggagaatgacaaaaaaaaaaaaaaaaaaaaaaaaaaaaaaaaa

The mCherry sequences are highlighted in red, the P2A sequences are highlighted in yellow, the strep tag sequences are highlighted in green, and the N sequences are highlighted in blue.

**Figure S1. *In vitro* stability of rSARS-CoV-2 mCherry-SN.**

(A) Plaque morphology of rSARS-CoV-2 mCherry-SN in Vero E6 and A549-hACE2 cells.

(B) Stability of rSARS-CoV-2 mCherry-SN after nine serial passages in Vero E6 cells. Plaques from passages 1, 5, and 9 are shown in A549-hACE2 cells.


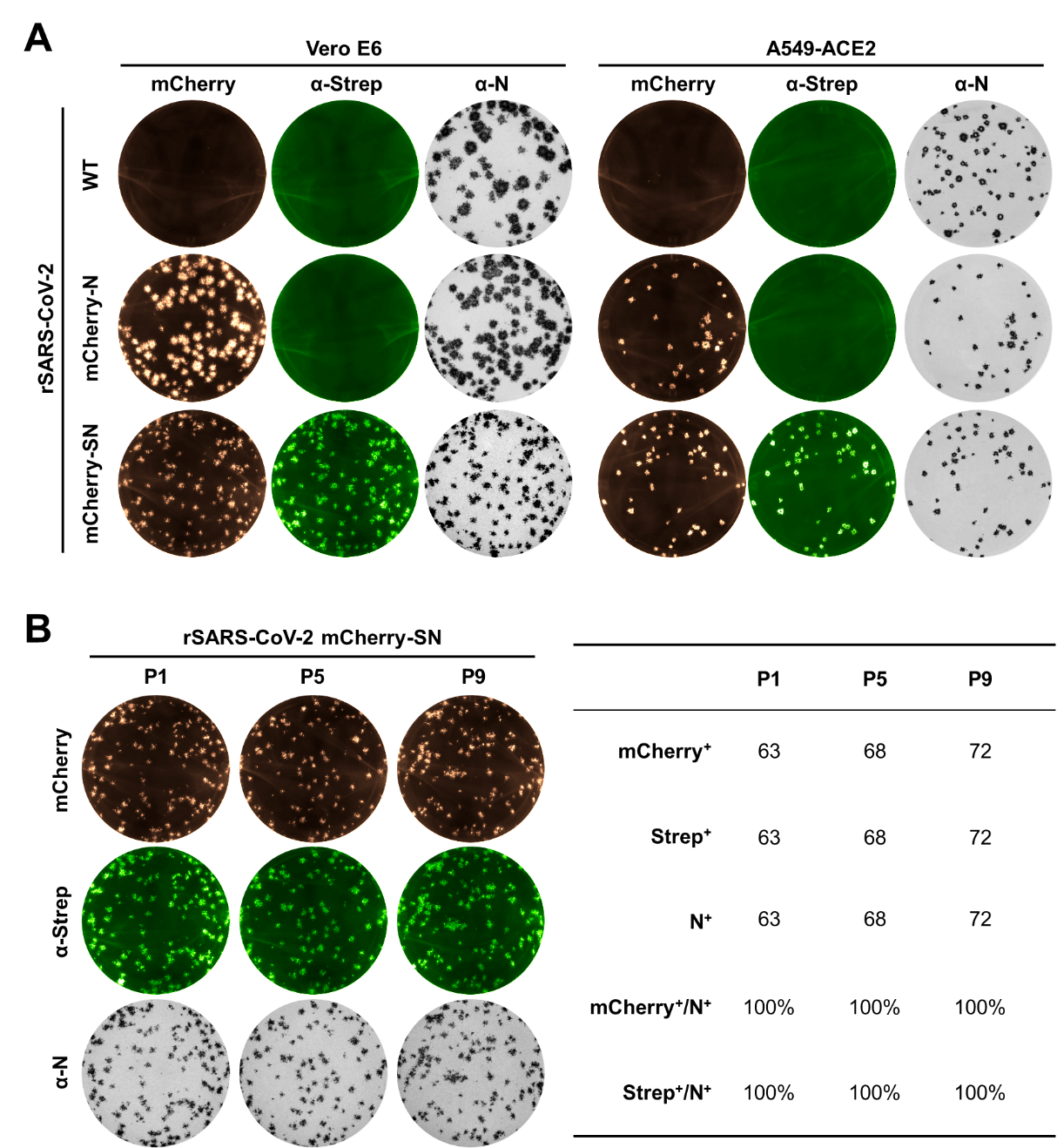


**Figure S2. Evaluation of Strep affinity purification samples.**

(A) SDS-PAGE and Coomassie Brilliant Blue (CBB) staining of samples from Strep affinity purification. Lanes 1 and 7: cell lysates; lanes 2 and 8: wash 1; lanes 3 and 9: wash 5; lanes 4 and 10: elution 1; lanes 5 and 11: elution 2; lanes 6 and 12: elution 3. (B) Western blot analysis of the same samples from panel A.


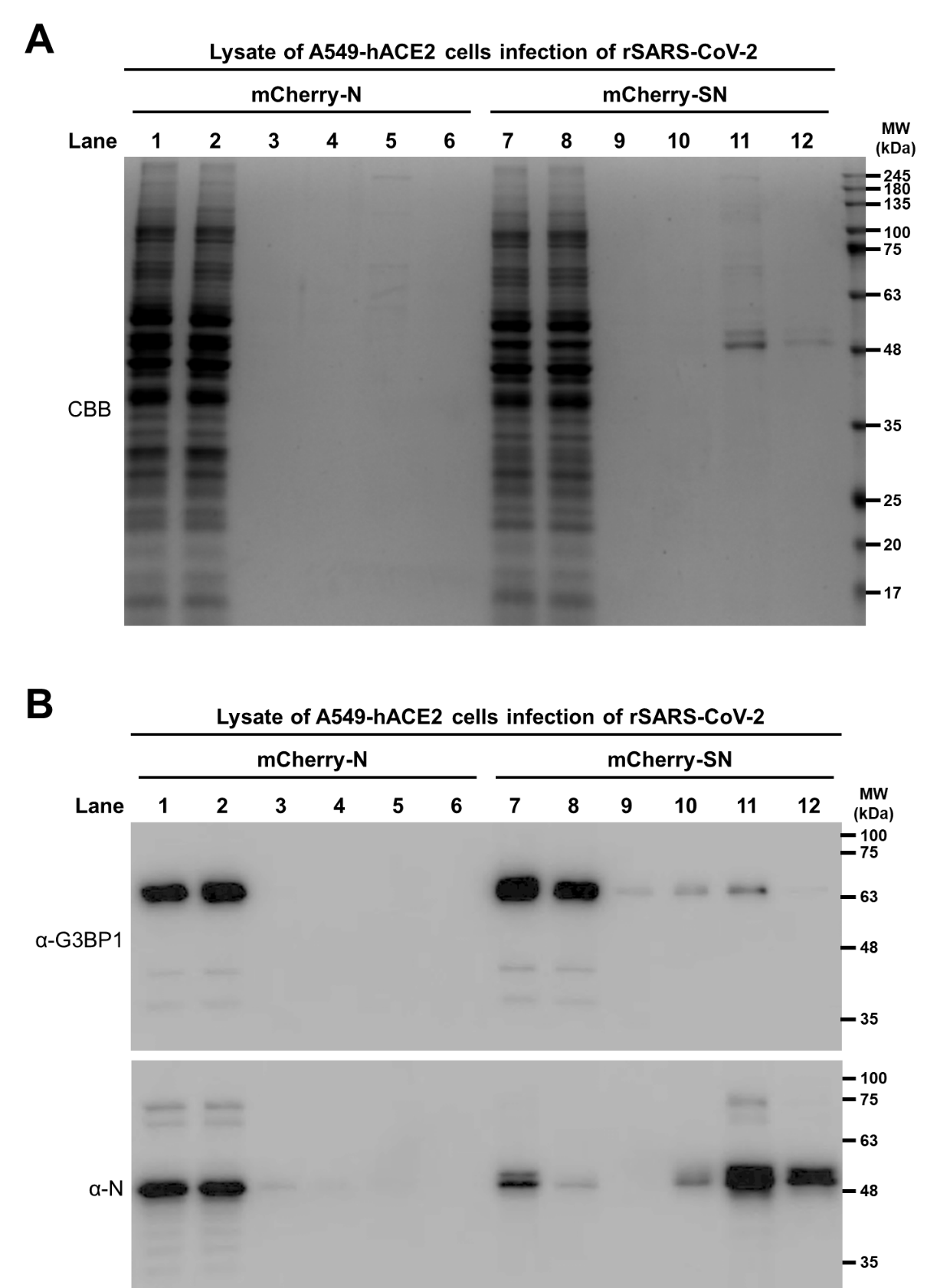


**Figure S3. Unique viral peptides identified in the Strep affinity purification samples by mass spectrometry.**

Unique peptides identified by mass spectrometry corresponding to other viral proteins.


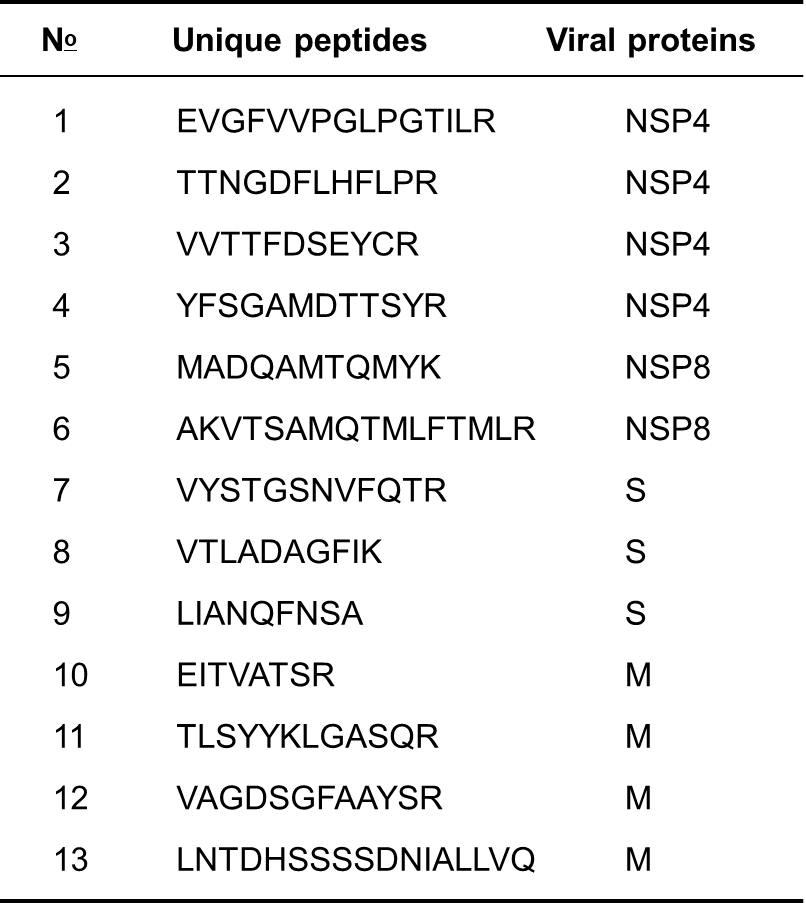

Supplement: Supplemental material — Viral sequences data; Fig. S1 to S3. [file spectrum.02915-25-s0001.docx]
